# Opportunities for Bio-Based Solvents Created as Petrochemical and Fuel Products Transition towards Renewable Resources

**DOI:** 10.3390/ijms160817101

**Published:** 2015-07-28

**Authors:** James H. Clark, Thomas J. Farmer, Andrew J. Hunt, James Sherwood

**Affiliations:** Green Chemistry Centre of Excellence, Department of Chemistry, University of York, Heslington, York YO10 5DD, UK; E-Mails: thomas.farmer@york.ac.uk (T.J.F.); andrew.hunt@york.ac.uk (A.J.H.); james.sherwood@york.ac.uk (J.S.)

**Keywords:** bio-based solvent, biomass, bio-refinery, green solvent, platform molecules

## Abstract

The global bio-based chemical market is growing in size and importance. Bio-based solvents such as glycerol and 2-methyltetrahydrofuran are often discussed as important introductions to the conventional repertoire of solvents. However adoption of new innovations by industry is typically slow. Therefore it might be anticipated that neoteric solvent systems (e.g., ionic liquids) will remain niche, while renewable routes to historically established solvents will continue to grow in importance. This review discusses bio-based solvents from the perspective of their production, identifying suitable feedstocks, platform molecules, and relevant product streams for the sustainable manufacturing of conventional solvents.

## 1. The Bio-Based Economy

The need for greener, sustainable chemicals has prompted a great amount of research into the processing of renewable feedstocks to obtain platform molecules and downstream end products [1,2,3,4,5,6,7]. Not only plant crops [8,9], but many varieties of wastes can be exploited to yield bio-based chemicals of interest; from the rinds of fruits [10], to the ashes of biomass incinerators [11]. Biomass as a feedstock to displace fossil resources has benefits relating to global carbon dioxide emissions, supply security, and projected long term economic savings [12]. The transition from fossil derived fuels and chemicals to renewable energy and bio-based products will have a profound impact on these markets.

Bio-based solvents are one class of highly sought after bio-based product [13]. Typical examples include limonene, ethanol, glycerol, and 2-methyltetrahydrofuran (2-MeTHF). A sustainable chemical industry will be reliant on the availability of renewable solvents, and much is being done at national and international regulatory and standardisation levels to facilitate the introduction of bio-based products, including solvents. In the European Union for example, a strategy for implementing and encouraging a bio-based economy has been launched [14], and a mandate issued specifically addressing the development of standards relating to bio-based solvents [15]. As a tool to support and enhance the bio-based economy, the purpose of standards is to increase market transparency and establish common requirements for products in order to guarantee certain characteristics such as a minimum value of bio-based content [16]. Bio-based solvents must also compete economically with established petrochemical solvents in order to gain a significant market share. The first output of this standardisation work, a technical specification for bio-based solvents, has now been published for the intended benefit of business-to-business (B2B) transactions [17].

**Figure 1:**
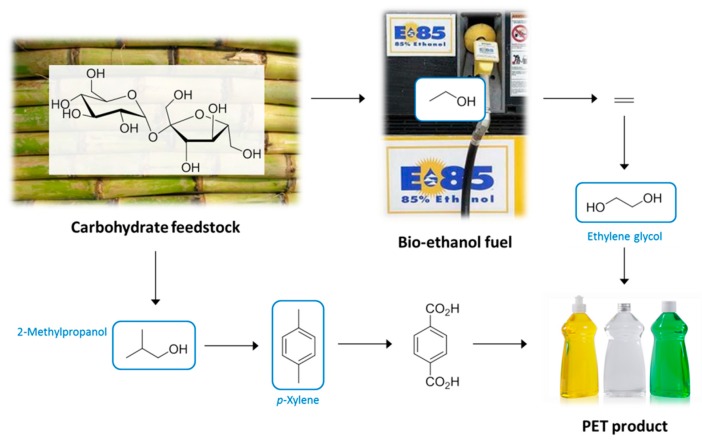
The interconnecting biofuel and bioplastic manufacturing industries and the solvent intermediates that result. Solvents are ringed in blue.

In this work the changing attitudes regarding solvent use will be put into the context of the increasing availability of bio-based solvents. The growing bio-based economy will generate a number of intermediate chemicals which will mostly be intended as a basis for the production of bioplastics. These so-called platform molecules will also find use in the production of solvents as their petrochemical equivalents do today. Greater volumes of biofuels has also created an opportunity for bio-based solvents, whether that be bio-ethanol and its derivatives, or vegetable oil products such as bio-diesel and glycerol. The potential of these feedstocks to be converted into bio-based solvents depends on market demand and legal restrictions on chemical hazards of course, but many of the solvents currently used in large volumes today can be obtained from either established biorefinery processes or from feasible chemical transformations of biomass proven at laboratory or pilot plant scale. This work will focus primarily on conventional solvents that already exist within the chemical production infrastructure. An illustrative example is the relationship between the bio-ethanol and bio-based polyethylene terephthalate (PET) industries (Figure 1). Solvents appear as the intermediates of the bioplastic production chain, with bio-ethanol itself a solvent. Whereas the neoteric bio-based solvents (*i*.*e*., those renewable solvents structurally dissimilar to conventional solvents) can have a limited market or issues relating to supply, purity, price, or undesirable properties such as high viscosity, the equivalent bio-based solvents featured in Figure 1 have established markets with proven demand. Neoteric bio-based solvents are viable products, but need to demonstrate their performance characteristics to gain support and develop a market share.

## 2. Contemporary Solvent Use

**Figure 2:**
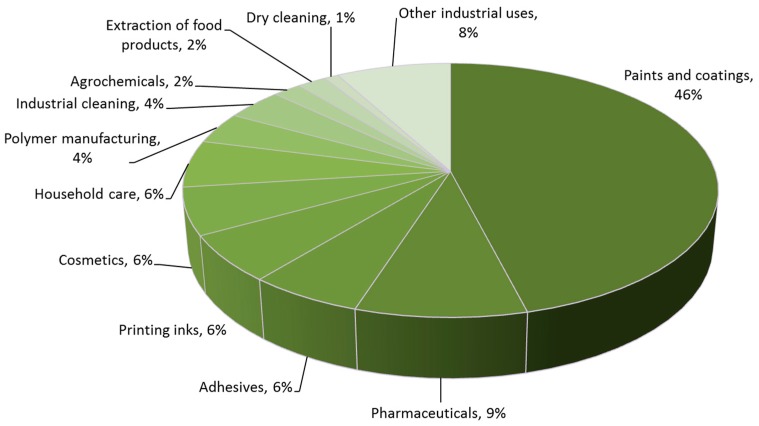
Solvent use arranged by sector.

The present day solvent market is on the order of 20 million metric tonnes (MMT) and worth tens of billions of US dollars annually to the global economy [18,19,20,21]. European solvent production provides about one quarter of the worldwide market [22,23,24], with annual bio-based solvent use in the European Union projected to grow to over one MMT by 2020 [25]. Important sectors include paints and pharmaceuticals (Figure 2) [26]. The solvent is often the major component of a formulation, a reaction, or an extraction [27]. As such a significant alleviation of non-renewable chemical dependence can be achieved with the implementation of bio-based solvents. The choice of solvent has a strong influence on the rate of reactions and substrate solubility, and the role of a solvent in a paint or coating formulation is different to that of a solvent used to facilitate the synthesis of an active pharmaceutical ingredient for example. Because of this, many different solvents are used across a variety of applications, and a large diversity of bio-based solvents is needed to relieve the present dependence on unsustainable fossil derived solvents.

At present solvent use is dictated by two influencing factors: regulation (compulsory) and organisational preference (not necessarily enforced). Regulations control the use of certain solvents and permissible residues in final products. This is true in the plant extraction industry for foodstuffs [28], and in the pharmaceutical sector for example [29]. Legislation has begun to rule out certain solvents from use completely because of environmental health and safety concerns. Benzene and carbon tetrachloride are two well-known examples of strongly regulated chemicals that were once popular solvents [30]. The present day tightening of chemical controls in Europe falls within the recently implemented Registration, Evaluation, Authorisation and Restriction of Chemicals (REACH) legislation [31], under which EU member states propose restrictions for chemicals with the potential to do harm. At the time of writing a number of solvents are on the list of substances of very high concern (SVHC), and restrictions on their use and import with limited authorisation will be considered. The solvents recognised as SVHC include amides such as *N*-methylpyrrolidinone (NMP) and *N*,*N*-dimethyl formamide (DMF), as well as some chlorinated solvents and certain ethers of ethylene glycol [32]. Solvents already subject to restrictions include benzene in products for public use, cyclohexane in neoprene-based contact adhesives, dichloromethane in paint strippers, the glycols ethers diethylene glycol monomethyl ether and diethylene glycol monobutyl ether (applicable to specific paint products), toluene (not to be used in spray paints), and 1,4-dichlorobenzene in air fresheners and similar products [33]. Chloroform, trichlorobenzene and several other chlorinated solvents are subject to wider ranging restrictions that forbid them from all products and substances intended for public use in concentrations greater than 0.1%.

After regulations have defined the set of solvents that may be used from a legal perspective, individual organisations can establish their own preference for certain solvents based on green chemistry principles [30,34,35]. Consumer pressure may also indirectly influence solvent choice, depending on the sector and how much “greenness” is valued [36]. One should also note that there can also be institutional resistance to change. This is not often due simply to stubbornness; because some processes and products must be registered, if the solvent was changed it would affect the purity profile of the product [29,37].

## 3. The Relationship between the Petrochemical Industry and Biorefineries

**Figure 3:**
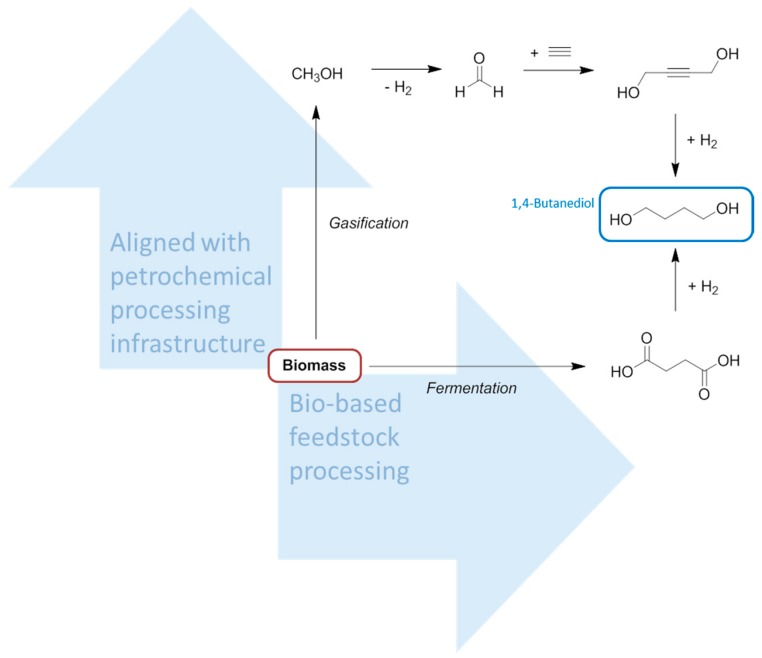
A comparison between the manufacturing processes of 1,4-butanediol from biomass. The solvent product is ringed in blue, the feedstock ringed in red.

Two options are available for the long term implementation of renewable chemical production: one is to abandon the centuries old techniques of the petroleum industry, making sometimes unfamiliar products with new technologies according to what suits the feedstock; the other option is to reduce biomass to hydrocarbons and phase this alternative feedstock into the pre-existing manufacturing structure (Figure 3), possibly as a blend of biomass and petrochemical feedstocks [38]. The latter takes advantage of low market barriers, while the former, more aspirational approach has been described as *“more visionary and will be harder to implement … owing to the fact that such potentially new commodity chemicals are not already part of the existing markets*” [12]. New processes also incur the financial penalty of startup costs and investment, whereas producing existing products using the same techniques but from biomass takes advantage of the existing infrastructure with the reassurance that a fossil derived feedstock can be relied on in case of price fluctuations or feedstock scarcity [39].

In reality there is not a clear cut decision between the two approaches and a combination of the two is beginning to occur. Commentary from academic sources seems to concur on extolling the value gained by embracing the chemical composition of biomass. In this work the other opinion is entertained, motivated by the need to produce bio-based solvents at realistic volumes and prices. The key role of the bioplastics and the biofuels industry in creating opportunities for bio-based solvents will also be explained.

There is a significant overlap between the chemical functionalities contained in bio-based solvents and conventional solvents. However the chemical basis of biomass feedstocks is different to crude oil and natural gas, and so many of the small molecules easily obtainable from biomass that could be considered solvents (or the precursors to solvents) will not necessarily always be equivalent to conventional solvents. For example, while bio-ethanol is indistinct from petrochemical ethanol, 2-MeTHF is not structurally identical to the solvent tetrahydrofuran (THF) it usually replaces, and the analogy is not necessarily this clear. Recent attempts to replace NMP and related amide functionalised dipolar aprotic solvents have made use of dihydrolevoglucosenone [40], and γ-valerolactone [41,42,43], neither of which contains nitrogen. For clarity, and given that the equivalent bio-based solvents have low market barriers, and the neoteric bio-based solvents are hampered by small production volumes, higher prices, and a limited number of suppliers, this review will limit its scope mostly to an overview of the bio-based feedstocks appropriate for synthesizing the conventional petrochemical base chemicals, emphasising the organic solvents that can then be obtained downstream. More direct reactions as in Figure 3 will be used for comparison, but the transition to a bio-based economy will have to rely on an integration of the petrochemical industry infrastructure with renewable feedstocks.

Through simple base chemicals such as ethylene, fossil feedstocks provide a lot of flexibility for chemical production. The additional benefit of tunable product streams reflects the historically variable demand for different types of fossil reserves and the downstream products that can be obtained (e.g., the water gas shift reaction yields more hydrogen from syngas at the expense of carbon monoxide, gasoline reforming converts alkanes to aromatics as required, inter-conversion between acetone and isopropanol (2-propanol) is balanced to match market demand). Conversely biomass conversion for chemical production might be regarded as less flexible because it generally generates highly functionalised products rather than easily diversifiable platform molecules. Instead it is the extensive variety of available biomass feedstocks that creates the prospect of a range of products. This situation can be thought of as both an advantage and a disadvantage for bio-based products. On the one hand, unique specialty chemicals made from biomass create opportunities in the niche high value chemical markets, but at the other end of the scale it may be difficult to provide replacements for many established commodity chemicals using renewable feedstocks when met with the challenges of economy and scale.

The technology of the oil refinery and the products formed bears little similarity to the current stage of development seen in present day biorefineries [44]. Whereas oxidation chemistry dominates petrochemical production, the primary products of a biorefinery are usually of a similar but not greater oxidation level to the feedstock (e.g., bio-diesel, fermentation alcohols), or reduced (e.g., furfural, isosorbide, levoglucosenone) [45]. The massive investment put into industries based on hydroformylation and oxidation act as an economic incentive to sustain these processes, and so reduction of biomass to hydrocarbons will remain attractive for decades to come, just so that the substrate can be subsequently oxidised. Roger Sheldon amongst others has warned against this rejection of the “redox economy” rule stating “*it is energetically more economical to avoid, as much as possible, changes in oxidation state during a multi-step process*” [46]. This is not always possible, for another justification for creating renewable hydrocarbons is to satisfy the substantial plastics industry where oxidation of the substrate is not applicable. Addition polymers including polyethylene and polypropylene dominate the plastic market [47]. Demand for bio-based polyolefins means the precursor monomers will also be available to feed into the solvent production chain.

**Figure 4:**
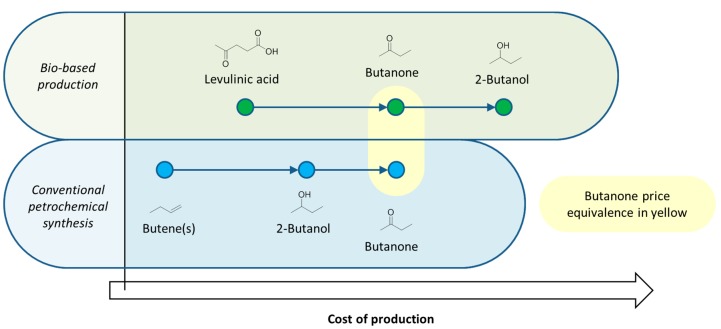
The effect of reversing the redox economy illustrated with 2-butanol. Relative costs of production have been estimated assuming a hypothetical scenario in which butanone prices are equivalent.

Occasionally oxidations are helpful in the primary conversion of biomass, such as in the synthesis of bio-based adipic acid for nylon. In the process developed by Rennovia [48,49], glucose is oxidised to glucaric acid, but to form adipic acid a quadruple reduction is then required using hydrogen gas [50]. An issue hindering a change from oxidation chemistry to reduction chemistry for the synthesis of bio-based solvents, even assuming the technological state of the art is comparable, is that the chemical market is well established and so are the prices, dictated by the dominant petrochemical supply chain. For example, butanone is more expensive than its precursor 2-butanol, reflecting the cost of the oxidation process (energy, additional materials, plant costs). If levulinic acid is decarboxylated to butanone [51], and that process made cost effective compared to the petrochemical approach to manufacturing butanone, then the downstream bio-based 2-butanol (as the product of reducing bio-butanone with hydrogen) will be more expensive than conventional 2-butanol and create a market barrier (Figure 4).

It is not only the direction of the change in oxidation state that distinguishes the refining of the two resources presently under discussion. Molecular complexity is increased by the processes of the oil refinery, adding an inherent value to the feedstock. A biorefinery will often apply processes to eliminate complexity (partial removal of heteroatoms notably oxygen, loss of chirality, *etc*.) because the intermediates used by the commodity chemical industry are for the most part shared, and must offer a broad basis of possible functionalisation. It has been argued previously that the functionality inherent to biomass resources should be used advantageously in the production of chemical products that reflect that functionality. One drawback to this philosophy already discussed is that markets for the resultant products are not established. Ultimately the issue is the cost of the biomass feedstock and the conversion processes not always being appropriate for the value of the chemicals our society requires in large quantities. Objectives for sustainable growth will also influence the scale of what a biomass producing or utilising enterprise can achieve, with land use issues and other sustainability criteria enormously important [52].

**Figure 5:**
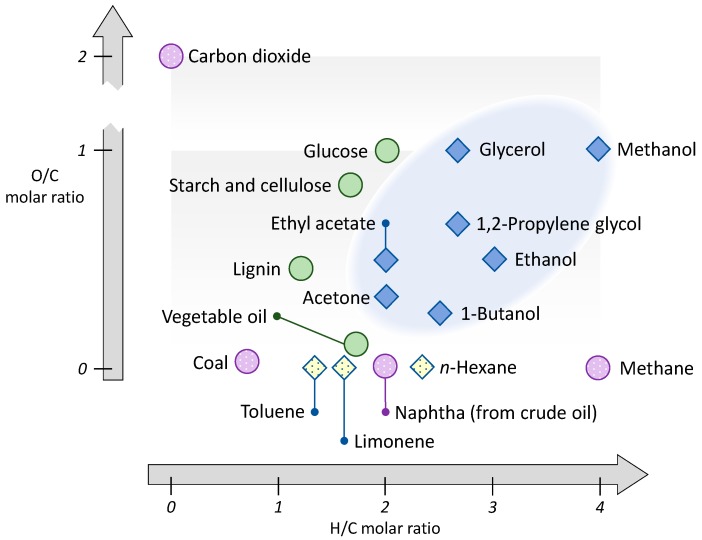
Chemical composition map of fossil derived resources (spotted circles), biomass feedstocks (solid circles) and representative organic solvents (hydrocarbons as spotted diamonds, oxygenated solvents as solid diamond).

The complexity of molecules can be crudely approximated by the number of heteroatom bonds. It is common in reviews describing biorefinery products to plot the molar ratios between carbon, oxygen and hydrogen as an indicator of the degree of functionality or energy of combustion [7,39,53]. These elemental ratios should not be confused as a measure of oxidation state because procedures such as a hydrolysis reaction can change the O:C ratio significantly without a change in oxidation state (glycerol liberated from vegetable oil by transesterification demonstrates this). Similarly oxidations that appear to impart a dramatic change in oxidation state do not have to represent a technologically difficult exercise. Conversion of methane to methanol (via syngas) is performed on an extremely large scale, producing a low cost product. Examination of Figure 5 shows that solvents reside in a region of relatively high H:C ratios with a mixture of O:C ratios. This is because solvents are usually small molecules with a single functional group, or on occasion two of the same functional group is present as observed in diols. Biomass feedstocks are similar in elemental composition, bordering the solvent region with slightly lower H:C ratios. Petrochemical feedstocks contain no oxygen, and are therefore closely related to their direct derivatives the hydrocarbon solvents. Carbon dioxide is an attractive feedstock but with limited chemistry options given its relative inertness.

In line with the perceived dissimilarity between the petrochemical industry and biorefineries, the relationships between bio-based solvents and equivalent products of the established petrochemical industry are quite varied. For example, acetic acid has mostly been produced from syngas using the Monsanto process (and subsequent refinements) since its development in the 1960’s, but to a lesser extent oxidative fermentation to acetic acid is also practiced, as it has been for centuries [54]. The process required depends on the application of the product, with many food uses only allowing bio-based acetic acid by law [55]. However the synthetic product is cheaper to produce so natural acetic acid only accounts of ~3% of the market [54,56]. This is an example of two quite different production methods yielding the same product. Tetrahydrofuran (THF) is being replaced by bio-based 2-methyltetrahydrofuran (2-MeTHF), made by the hydrogenation of furfural [57]. This process is also orthogonal to the chemistry of the petrochemical industry, and the final products are different, but the market and application as a solvent are the same. Relying on existing solvent markets provides the necessary entry point for this neoteric bio-based solvent.

As for the application of interchangeable feedstocks within petrochemical plants, ethanol is now used to directly synthesise ethyl acetate [58,59]. In any process where bio-ethanol is introduced in place of synthetic ethanol, no additional engineering efforts are needed to operate the process aside from routine moisture content and purity profile checks. The same applies to any analogous chemical upgrading of an intermediate with an alternative bio-based source.

At the base chemical level, co-gasification processes are now being operated with biomass and coal [60,61]. This means that downstream products will contain some bio-based content, although potentially variable, and ideally any solvents would be recognised as bio-based if their bio-based content was sufficiently high with a guaranteed minimum. Vegetable oil can be subjected to hydrocracking to produce liquid hydrocarbon fuels to replace diesel [62,63]. In a special instance, if castor oil is subjected to cracking a variety of unique products can be created; pyrolysis yields C_7_ and C_11_ products, while alkali splitting generates C_8_ and C_10_ products such as 2-octanol [64]. Therefore the choice to either conform to existing chemical market demands or exploit the novel attributes of biomass feedstocks presents itself once again.

**Figure 6:**
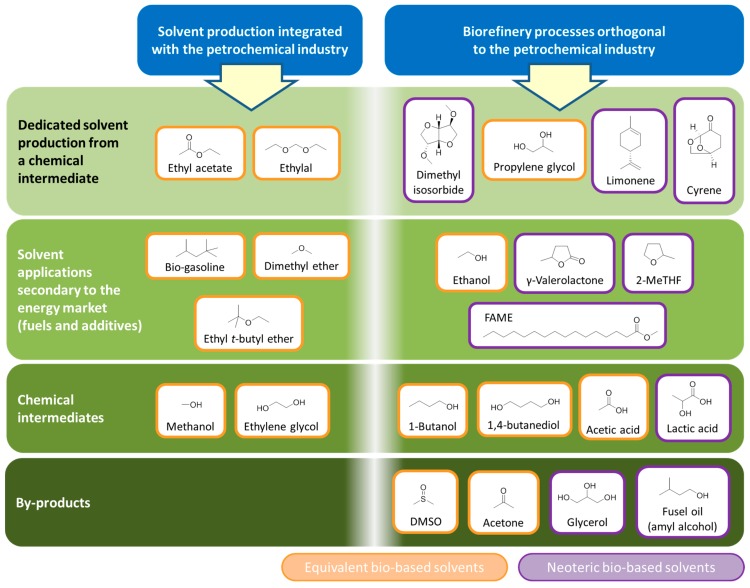
The relationship between different bio-based solvents according to their origin and their place in the chemical industry hierarchy.

An important consideration regarding bio-based solvents is the fact that several prominent solvents are either chemical intermediates (usually for the larger plastics market), or the by-products of processes. It can also be the case that these chemicals require one or more further chemical transformations to yield the actual solvent molecule. This goes some way to justify efforts to produce bio-derived platform molecules entering the chemical industry early in the production chain so that more downstream products stem from the biomass feedstock. An example is bio-ethanol, now dehydrated to ethylene for the production of plastics (*i*.*e*., polyethylene and PET) [65,66]. The intermediate monomer of bio-based PET production is ethylene glycol (Figure 1), which also has applications as a solvent (derived from bio-ethanol or otherwise). Conversion to competitively priced glycol ether solvents then becomes feasible given the bio-based monomer is made available in large quantities. Similarly with the bioplastic polylactic acid (PLA) now occupying a share of the plastics market [67], the intermediate lactic acid (and its esters such as ethyl lactate) have become available on the market as neoteric bio-based solvents [68]. Acetonitrile is a by-product of acrylonitrile manufacturing, which means the feedstock would have to be changed to bio-propylene to create a supply of bio-based acetonitrile by the usual means.

The remaining solvents (aside from those few solvents that are valuable enough to demand dedicated production) are closely linked to the manufacturing of liquid biofuels. Ethanol is the primary example of a biofuel that is also a solvent. Other bio-based solvents are likely to occur as value adding co-products of fermentations, such as the acetone formed in bio-1-butanol fuel production [69,70]. Solvents derived from the glycerol of bio-diesel production have been investigated extensively but they are structurally very different to conventional solvents and may not achieve an important market share [71].

Not dissimilar to previous assessments [39], this discussion leads to the definition of four categories of bio-based solvent, which are summarised in the following pictorial representation (Figure 6). This ordering is further divided into equivalent and neoteric bio-based solvents produced within either of the petrochemical or biorefinery models. An economic hierarchy can be developed, with solvents arising as by-products and intermediates possibly more economically attractive (e.g., glycerol) but supply is very much dependent on a potentially fluctuating demand for the major product (*i*.*e*., bio-diesel in the case of glycerol). Nevertheless neoteric solvents made by novel biomass processing are viable products, despite the price difference compared to conventional alternatives. Limonene is primarily obtained as the major fraction of citrus oils [72,73]. Conversely the racemic dipentene is synthesised from α-pinene as a consequence of terpineol production, and would be considered as a by-product [74].

## 4. Production of Petrochemical Intermediates Relevant to Solvent Manufacturing

Crude oil production was four billion metric tonnes in 2013 while natural gas production exceeded three billion metric tonnes (on an “oil equivalents” energy basis) [75]. Recent increases in shale gas production have meant the extraction of this resource now exceeds that of gas obtained from conventional sources in the USA [76]. Crude oil is the conventional feedstock for liquid fuels, with Fischer-Tropsch technology also contributing via the transformation of syngas originating from natural gas and also coal [77]. Biomass is already used as a feedstock in gasification plants at scales relevant to feed into the Fischer-Tropsch infrastructure, and may help reduce the large greenhouse gas emissions associated with the coal-to-liquids industry [78,79,80,81]. Petrochemical production constitutes less than 4% of crude oil use, but this derives almost half of its downstream value [39,53].

**Figure 7:**
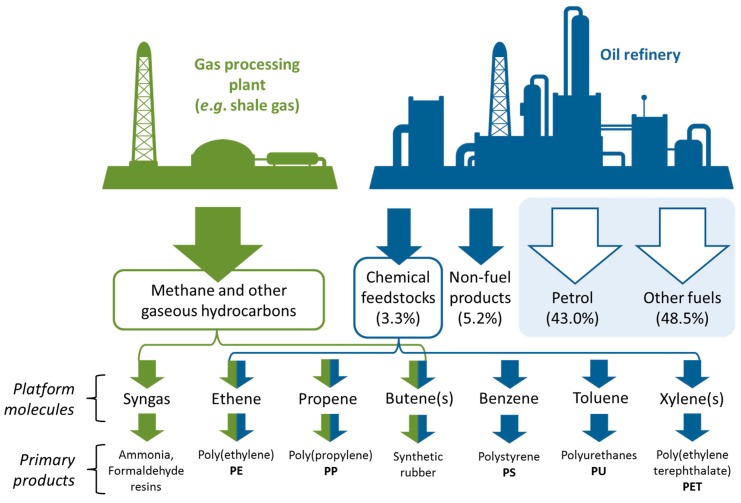
The primary fossil resource refining product streams.

As a hydrocarbon mixture, functionalisation of crude oil derivatives is achieved through the addition of oxygen and other heteroatoms. However the effort required to prepare the hydrocarbon substrates suitable for this further processing should not be overlooked. For fuel and chemical production crude oil is desalted before being distilled at 400 °C [82]. Fractional distillation creates the cuts of petroleum needed for different products, and vacuum distillation is required for the heavier fractions. Further refining by thermal or catalytic cracking, hydrocracking, isomerisation and even solvent extraction are used to transform the appropriate fractions into recognised chemical streams. Crude oil is supplemented by natural gas and coal as the basis for the production and isolation of methane, alkenes (C_2_–C_4_), and aromatics collectively known as BTX (benzene, toluene, and the xylenes) as shown in Figure 7 [82,83]. These are the so-called base chemicals from which petrochemical products are made. The most important products in terms of market size are plastics with 265 million metric tonnes produced in 2010 [18,47].

The petrochemical base chemicals provide the necessary intermediates for the vast majority of conventional organic solvents, often with the primary purpose of producing plastics. By creating the bio-based analogues of these platform molecules, all organic solvents could become available from renewable sources, produced using the existing chemical industry infrastructure. This includes chlorinated solvents. Although possible to produce from biomass feedstocks, no significant discussion of the halogenated solvents will be presented in this work given their undesirable health and environmental impact, even though they possess attractive physical properties as solvents. Replacing halogenated solvents proved to be difficult in the wake of tightening regulation [84], but looking beyond compulsory substitution, a consensus that replacement is preferable even when legislation does not directly influence that decision created a demand for alternative solvents that can operate in this role [85,86,87,88]. Broadly speaking, these efforts fall within the wider context of air pollution control, which continues to evolve since some chlorinated solvents were identified as ozone depleting in the 1980’s [89,90].

The types of fossil derived hydrocarbon that are primarily produced for fuel purposes also find use as solvents, notably *n*-hexane for extractions. Interest in creating direct bio-based substitutes for gasoline and diesel has yielded commercial results. The American company Virent have developed a process of turning carbohydrate into gasoline hydrocarbons [91,92,93], and Neste Oil produce a renewable diesel from vegetable oil (not fatty acid methyl ester bio-diesel but a hydrocarbon mixture created by hydrogenation of vegetable oil) [94]. These fuel products could be distilled near the end of production to yield more discrete chemical fractions suitable as solvents, although this is not being done in practice. Alternatively, other hydrocarbons not necessarily currently associated with liquid fuels can be produced from biomass. The alkene *trans*-β-farnesene is produced commercially by Amyris [95]. Recently academic work has shown the synthesis of methylcyclopentane is possible via 5-hydroxymethylfurfural (HMF) [96].

The remainder of this review will address the solvents derived from the base chemicals obtained as the chemical feedstock stream in Figure 7. The alkene base chemicals are used to produce the “oxygenated solvents”, called as such to distinguish them from the hydrocarbon and chlorinated solvents mentioned in passing above. This is also true of methane when not fed into the energy sector. The BTX chemicals also have fuel applications in addition to their role as chemical intermediates. These aromatic base chemicals have fewer applications as solvent precursors. Instead they are mostly used as solvents directly and this is reflected in the following discussions. Finally, a concise comparison to neoteric solvents is made, briefly exploring the synthesis of these solvents without the need to go via any of the recognised base chemicals, and what role this will have in future solvent manufacturing and use.

## 5. Methane and Syngas

The argument for replicating the petrochemical base chemicals from biomass resources in order to maintain the existing chemical industry infrastructure is strongest when the available biomass resource is a mixed waste (e.g., general food supply chain waste). In this situation the only broadly applicable and economically viable process that is likely to extract value in the form of a chemical intermediate will be anaerobic digestion. This is because anaerobic digestion plants require relatively little investment coupled with low energy consumption [97]. Estimates for the amount of biomass available for anaerobic digestion within the EU amount to 1500 million tonnes per year [98].

The product of anaerobic digestion is biogas, consisting of methane and carbon dioxide. Anaerobic digestion is the only chemical producing food waste treatment currently in operation in the UK (however it only accounts for 1% of food waste utilisation) [99]. That is not to say that mixed food waste is not able to be converted into other products. Recent research just from the year 2015 indicates a broad spectrum of processes may be applicable in the goal to help eliminate food waste as a concern and instead upgrade it into a valuable resource (Table 1) [100,101]. Other base chemicals in the form of aromatic compounds (benzene, toluene, *etc.*) can be obtained from mixed food waste when pyrolysed in conjunction with agricultural waste [102]. Regarding high value chemicals, it is more common to obtain these products from the oils of specific food waste streams (e.g., coffee grounds [103], or citrus peel [104]).

**Table 1:** Recent process aimed at converting mixed food waste into chemical intermediates.

Process	Product	Reference
Anaerobic digestion	Methane	[105,106]
Sludge reactor	Hydrogen	[107]
Fermentation	Ethanol	[108]
Fermentation	Acetic acid	[109]
Fermentation	Lactic acid	[110]
Dehydration	5-hydroxymethylfurfural (HMF)	[111]
Catalytic fast pyrolysis	Liquid aromatic hydrocarbons	[102]

The use of a methane as a fuel is helpful for the economic viability of the anaerobic digestion process. It establishes a reliable market and justifies large-scale production, in turn reducing manufacturing costs to the benefit of secondary uses as a base chemical. At present methane is primarily obtained as the major component of natural gas. This resource is often found in oil fields and released upon depressurisation of the crude oil reserve during commercial drilling operations [112]. Increasingly methane is now obtained from shale rock (fracking), again with the primary purpose of providing a source of low cost fuel, although the recent drop in crude oil price beginning in 2014 has put this strategy into doubt [113]. As an equivalent chemical product to natural gas and shale gas methane, biogas is already incorporated into the energy sector on a localised scale [114], and notably as an important part of the German national energy network [115]. The actual process of anaerobic digestion, by which low value crop residues and food waste are converted to bio-methane by anaerobic microorganisms is complex and beyond the scope of this review, but descriptions can be found elsewhere [98,116,117]. What is important to recognise in the context of this work is that the feedstock options for anaerobic digestion are broad (although lignin is not digested) and the operation scalable within the biorefinery concept.

Methane is an established source of downstream chemical products in addition to its obvious use as a fuel. The key, initial stage is the splitting of methane into syngas (carbon monoxide and hydrogen gases) by steam reforming, providing an invaluable platform onto countless other chemicals that are of interest as solvents and other functional chemicals [118,119]. Alternatively gasification processes can be applied to biomass to directly produce syngas without the need for biological treatments or the intermediate methane [120]. This may be most helpful for the valorisation of lignin [121,122], for lignin is not processed during anaerobic digestion and is generally difficult to convert into other chemicals. This includes the lignin-rich black liquor of the pulp and paper industry as a low value resource in need of valorising [123].

**Figure 8:**
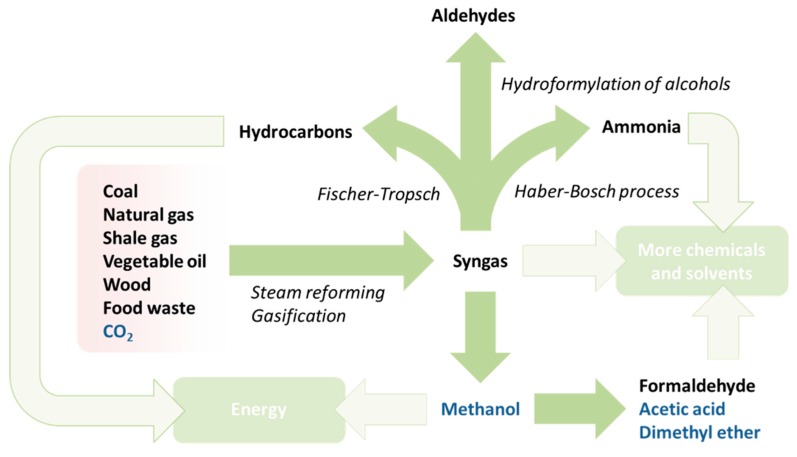
The extended syngas and methanol economy, with feedstocks highlighted in red and solvents in blue text.

Syngas is required for hydroformylation chemistry to convert alkenes into alcohols. In the case of the Fischer-Tropsch process syngas is also utilised, but for the production of hydrocarbons [77], thus establishing a strong precedent for a potentially large library of chemicals to be produced from bio-methane. It is also worth drawing attention to the carbon dioxide co-product of anaerobic digestion, which can be compressed to create a supercritical solvent of great utility in extractive processes without the toxicity issues of hydrocarbons like n-hexane [124,125,126,127]. After the initial feedstock refining, the primary use of syngas is for the production of methanol [128]. Hydrogen gas has another immensely important role in the formation of ammonia by the Haber-Bosch process [129]. These simple transformations of methane, affording carbon monoxide and hydrogen, onto methanol and ammonia in tunable proportions, provides access to enormously important chemicals from which a variety of solvents are made (Figure 8).

Methanol of course is a solvent in its own right, although its toxicity is an issue [130]. As of 2013, 0.2 MMT of bio-methanol was being produced annually [131], contributing 0.4% of the total methanol market share. Bio-methanol is presently produced from the gasification of wood, the black liquor from paper pulping, glycerol, and carbon dioxide, and in all cases often hampered by production costs. This is mainly because the scale is not suitably large yet to justify the capital cost [131]. Larger production plants are in development, with dimethyl ether an interesting biofuel product along with the usual plastics industry uses for methanol [132]. When the gasification of biomass is combined with the synthesis of methanol in a single process it provides a useful product but limits the downstream chemistry. Carbon monoxide is still needed by the chemical industry for other reactions, as is hydrogen gas. For this reason syngas will be regarded here in this work as the key intermediate for solvent synthesis, not methanol.

**Figure 9:**
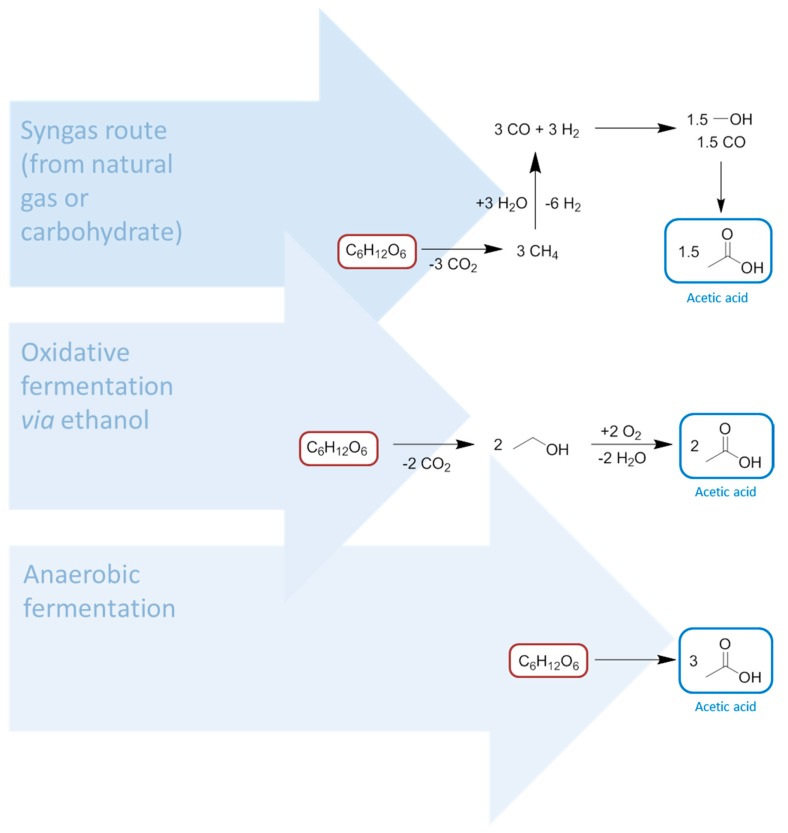
Stoichiometric reaction schemes for three approaches to the biosynthesis of acetic acid.

Regarding solvent products downstream of methanol, carbonylation to give acetic acid from methanol is the industry standard procedure for this product (Figure 9) [56]. Bio-based acetic acid is made by fermentation but on a much smaller scale [54]. Here is an example where present day economics (guided by historical precedent) means the favoured process for production of bio-based acetic acid is not analogous to the petrochemical route despite the availability of bio-methanol, albeit in relatively small quantities. Current progress would suggest that it is unlikely that acetic acid made by bio-methanol carbonylation will become more important than fermentation strategies due to this historical precedent. Typically it is an oxidative fermentation process from ethanol that gives bio-based acetic acid and is the basis of making the foodstuff vinegar [133]. This traditionally important process should be supplanted in time by a direct anaerobic fermentation of cellulosic sugars boasting 100% carbon utilisation [4]. This should be compared to the equimolar co-production of carbon dioxide observed during the fermentation to ethanol (Figure 9). Anaerobic digestion proceeds through an acetate intermediate, and the adaptation to stop at an acetic acid end-point significantly shortens the chemical synthesis pathway compared to the complete reduction of biomass to methane and then subsequent oxidation back to acetic acid [134]. This type of synthetic shortcut, often provided by fermentation, creates specificity at the expense of the broader diversification potential that a base chemical provides. The recurring question of whether to utilise the functionality of bio-based products or re-join the conventional chemical industry infrastructure using hydrocarbon base chemicals would seem to be more easily answered when the target molecule (acetic acid in this example) is actually an intermediate in the process of producing its conventional precursor methane.

Ultimately the question of which approach to renewable acetic acid production to adopt is answered by economic arguments, but this is complicated by the price of commodity chemicals being prone to fluctuation. From a mass balance perspective anaerobic fermentation would appear to represent an optimal process. A simplified assessment, through estimates of the energy required to produce chemicals, gives an indication of market competitiveness. Cumulative energy demand (CED) is also recognised as a useful approximation of the environmental burden (cradle-to-gate) of a process [135]. Production of acetic acid by the carbonylation of methanol is a relatively low energy process (55 MJ/kg) in the context of solvent production [136]. Additionally it is worth noting that because roughly 25% of the methane input for steam reforming is simply burnt to maintain the 600 °C reaction temperature, about 0.6 kg of carbon dioxide is released per kilogram of acetic acid produced [12].

**Figure 10:**
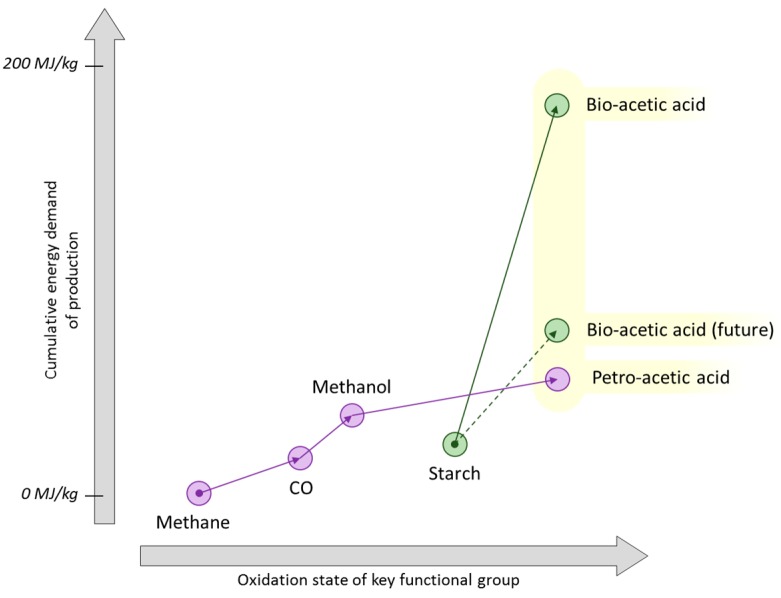
An indicative diagram plotting cumulative energy demand as a function of changing oxidation state for the production of acetic acid.

Compared to methanol carbonylation, the present day batch anaerobic fermentation process to give acetic acid directly from carbohydrate is not competitive in terms of energy utilisation (Figure 10) [54,136,137]. In this diagram each cumulative energy demand (CED) is given as a function of the highest carbon atom oxidation state present at a relevant functional group (on a qualitative basis). The *x*-axis of Figure 10 demonstrates the degree of chemical modification is not related to the energy requirements of the process, calling into question the usual proposition of “redox economy”. Thus maintaining the high oxidation state and functionalisation of a biomass feedstock is not a guarantee that the production energy requirements are minimised. Fermentations typically require energy intensive separations that are not obvious from the balanced chemical equation describing the synthesis, and this has a large influence on the CED. It is anticipated that a continuous flow fermentation would bring the CED of the production of bio-based acetic acid to within a reasonable comparison of the petrochemical route, as indicated by the dashed line in Figure 10 [54].

Acetic acid is not a common solvent, mostly used in the synthesis of vinyl acetate for polymer applications [56]. It has some limited uses as a solvent, such as for terephthalic acid purification, and in an indirect way as a reactant in the synthesis of ethyl acetate and other esters [54]. The esters of acetic acid are valuable solvents for extractions and formulations. Methyl acetate is a co-product of the synthetic methanol Monsanto and Cativa carbonylation processes, and a chemical intermediate in its own right (in this regard it is mostly used as a feedstock for acetic anhydride production) [138]. Most other esters of acetic acid are generally produced by Fischer esterification, although ethyl acetate can be manufactured from ethanol as the sole reactant [139,140]. Process intensification through reactive distillation means the number of possible combinations of fermentation alcohols and carboxylic acids makes for a large catalogue of easily accessible bio-based ester solvents.

**Scheme 1:**
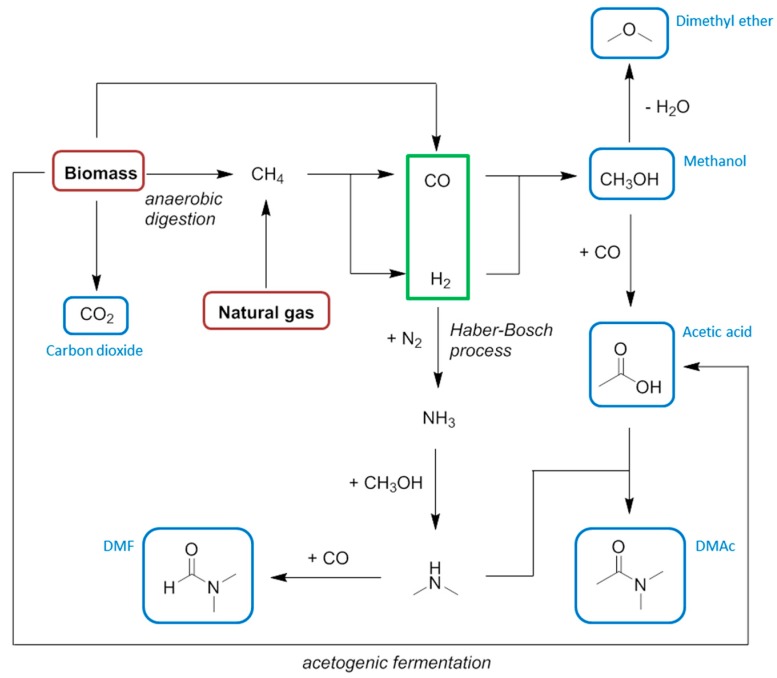
The direct solvent derivatives of methane and syngas. Key: red = feedstocks, green = key intermediate, blue = solvents.

There are further carbonyl containing solvents comprised of syngas derivatives, notably the amides DMF and DMAc (*N*,*N*-dimethyl acetamide, Scheme 1). Another highly dipolar aprotic solvent, DMSO, can also be produced from methanol via dimethyl sulphide, but in reality this intermediate is obtained from the Kraft pulping process and oxidised to (bio-based) DMSO [35]. Present day DMF and DMAc manufacturing plants are based on non-renewable feedstocks. The ability to make these solvents from bio-gas in the near future could be perceived as important to the synthetic chemist because they provide the ideal medium for cross-coupling reactions as well as other chemical transformations that require a highly dipolar and aprotic medium [141]. The realisation of this scenario will almost certainly be prevented by legislation correctly identifying most amide solvents as being toxic for reproduction, including DMF and DMAc [32].

**Scheme 2:**
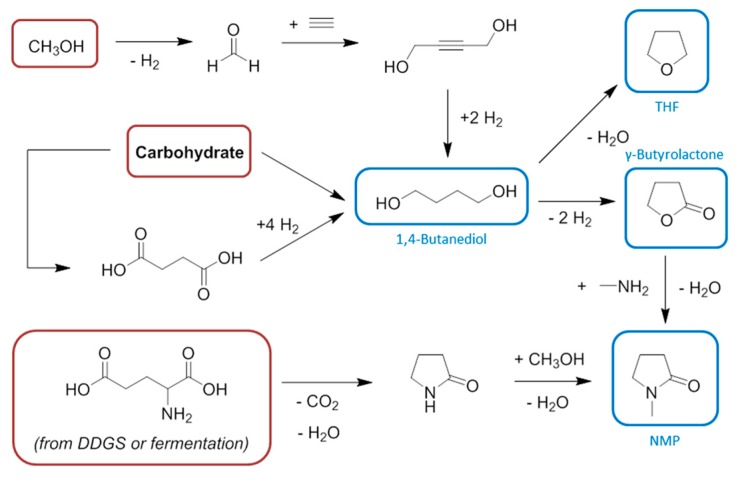
The synthesis of bio-based solvents conventionally produced from methanol. Key: red = feedstocks, blue = solvents.

As is the case for DMF and DMAc, both THF and NMP are manufactured from syngas and methanol (Scheme 2). Although this emphasises once again the potential of bio-derived syngas to be integrated into the future solvent manufacturing infrastructure, the key intermediate of both the THF and NMP syntheses, 1,4-butanediol, can be obtained directly by fermentation [142]. However given the status of present day technology it is more attractive to obtain 1,4-butanediol from the reduction of bio-based succinic acid, also a fermentation product [4]. 1,4-Butanediol is a solvent in its own right [143,144,145], although in this regard it receives limited attention compared to its application as a monomer for the plastics market [146,147]. It is obvious that starting from the base chemicals results in is a longer synthesis of THF or NMP than the shortcut provided by fermentation to give the intermediate 1,4-butanediol. It is also useful to note that succinic acid can be reduced and dehydrated to THF in a single step, circumventing the need to isolate 1,4-butanediol and further proving the established synthetic routes are not essential [148]. This argument is equivalent to the state-of-the-art relating to acetic acid production, best produced directly by the anaerobic fermentation of carbohydrate and not from bio-derived syngas. This of course requires the pull of the biofuel and plastics sectors to justify the specific synthesis of acetic acid on the correct scale rather than the more widely useful syngas.

It is also worth considering other feedstock options, and glutamic acid for the synthesis of NMP and related nitrogen containing compounds is gaining attention [149,150,151,152]. Glutamic acid is found in dried distillers grains with solubles (DDGS), a by-product of brewing and biofuel production [153]. The DDGS consists of 20–30 wt % protein, which in turn is typically 12%–20% glutamic acid, the most prevalent amino acid in this waste stream [154,155,156]. Decarboxylation provides access to the pyrrolidinone moiety via 4-aminobutyric acid. Ultimately the feedstock option selected by a chemical manufacture will depend on many factors, including availability and price. As more opportunities to convert biomass into chemical products are developed, what is presently considered to be low value waste can easily become a highly valuable commodity [39]. Thus the dedicated fermentation to give glutamic acid may remain preferable [157], or of course the conventional (and certainly cheaper at present) route based on bio-methanol preserved.

As is true of the other common amide solvents, the reproductive toxicity of NMP means legislation (REACH) will prevent its general use in the near future [32], bringing the usefulness of this procedure into question. Investigations into benign replacement dipolar aprotics are underway, although bio-based solutions are scarce at present [40]. When toxicity is an issue for a solvent, development of a green alternative is unlikely to be successful by making minor adjustments to the structure of the solvent or by changing the feedstock.

Because conventional solvents tend to be based on the petrochemical base chemicals, and toxicity is related to chemical structure, it could be argued that we must embrace different molecular templates and modify them to produce unconventional, neoteric solvents without the familiar toxicity issues, and even improved physical characteristics. Biomass presents an ideal opportunity to do this, and forms the crux of the usual fossil versus biomass feedstock debate. This approach is demonstrated well with the two solvents tetrahydrofuran and NMP featured in Scheme 2. Academic exercises have sought to replicate the performance and characteristics of *N*-methylpyrrolidinone with other solvents, ideally made from a renewable source. Amides of levulinic acid have been suggested as potential solvents for this purpose [158]. Commercial (and pre-commercial) NMP replacement products are also available, such as TamiSolve NxG [159] and Cyrene [160]. Similarly THF can be replaced by bio-based 2-MeTHF, which offers superior water separation [161]. None of these substitute solvents are primarily derived from synags, although its hydrogen component remains vital for the production of these solvents.

To summarise the methane platform, it is clear that in fact is it syngas that is the crucial intermediate for making solvents. Biomass offers a variety of ways to obtain syngas or methanol directly that do not rely just on the steam reforming of methane, although economic arguments highlight a market barrier [46]. Gasification of a large choice of feedstocks will contribute to future syngas and methanol production. Also noteworthy are fermentations using syngas as the feedstock [46,162], where butanol and hexanol are end products [163]. Higher alcohols still (particularly 1-octanol) can be obtained using genetically engineered bacteria, but fed on a conventional glucose feed [164]. Alternative technologies for hydrogen (fuel) production can also make a relevant contribution to the chemical industry, including algae bioreactors, [165,166,167] and water splitting [168]. An anticipated future scenario in which increasing amounts of bio-based methanol is produced will inevitably mean some will end up contributing to the synthesis of solvents downstream in the chemical manufacturing industry, and possibly on a significant scale. ‘Methanol-to-olefins’ conversion technology will maintain interest in bio-based methanol [169,170], despite the synthetic shortcuts to the downstream solvent molecules described previously. A methanol economy grounded in carbon dioxide capture is another motivation [171,172,173], although bio-methanol has been overshadowed within the biofuel sector by advances in bio-ethanol, which is strongly linked to the production of ethylene, the next of the conventional base chemicals to be addressed.

## 6. Ethylene

Unlike the production of biogas to supplant natural gas, replacement of the heavier hydrocarbon base chemicals with bio-based alternatives is not so immediately integrated or necessarily complementary from a technology perspective. It is a challenge for most sources of biomass to compete with naphtha as a chemical feedstock, which has an ideal chemical composition for fragmentation to olefins, including ethylene, which enjoys the largest market share of the olefin base chemicals [39]. Routes to renewable olefins typically rely on the dehydration of fermentation alcohols. Bio-based ethylene is produced in this way [65,66], as it was historically [12]. The process operates counter-current to the conventional petrochemical production of ethanol by the hydration of ethylene, a process worth noting as nearing redundancy due to the mass adoption of bio-ethanol as a fuel [54].

It is important for the bio-ethanol market that this fuel can be produced in a cost competitive way, which presently relies on subsidies and the push of renewable energy quotas [174,175,176,177,178,179]. Going deeper into the chemical value chain, where bio-ethanol is being used to access ethylene and subsequent downstream solvent products, their price will not be competitive if the ethanol prices (bio-based and fossil derived) are comparable. Bio-ethylene is downstream of ethanol, whereas in the oil refinery petrochemical ethylene comes before ethanol in the production chain and is therefore cheaper.

**Figure 11:**
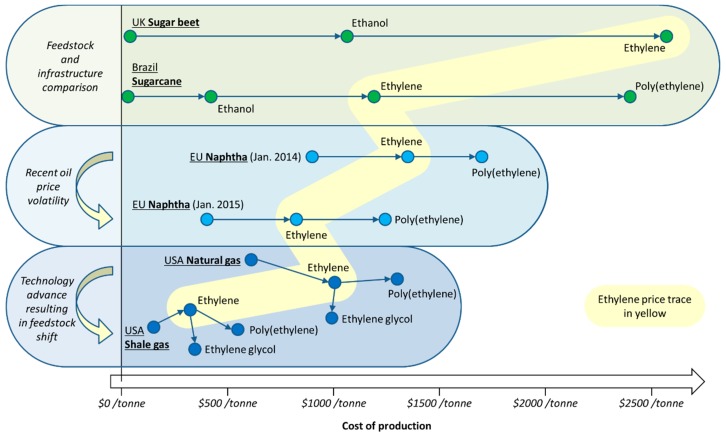
Cost diagram for ethylene feedstocks and representative downstream products [180,184,185,186,187,188,189,190].

Some data is available to help discern the ability of bio-based ethylene to provide a viable alternative feedstock. Estimations for the development of an ethylene manufacturing process using sugar beet from Europe puts the cost too high to be competitive for a commodity product [180]. Conversely the use of Brazilian sugarcane ethanol as a feedstock for ethylene, commercialised by Braskem in 2010 [181,182], was competitive with petrochemical ethylene in 2014 (Figure 11). Unfortunately the scale of the operation with its higher manufacturing costs restricts the final bio-based polyethylene product to premium applications, with finished product prices possibly as much as 30% higher [180]. Recently the economics relating to the production of rival fossil feedstocks could threaten progress in the growth of bio-ethylene production. One issue is the volatility of crude oil prices [183]. As of January 2015, the cost of producing ethylene from naphtha had dropped below that for bio-ethylene. Thus bio-based polyethylene, one of the vital drivers for sustaining bio-ethylene production and therefore permitting the production of ethylene derived bio-based solvents, is, at the time of writing, exposed to the economic drivers of the chemical market. This applies equally to downstream products such as ethylene glycol (solvent and monomer in PET production).

In the long term the price of crude oil relative to biomass feedstocks is anticipated to generally increase [12], and as the technology and infrastructure of bio-ethanol processing improves and expands, the price of bio-based ethylene should fall. Considering the ambitions of Braskem for example and the governmental incentives in place for the bio-based economy, bio-ethylene derived plastics to a lesser or greater extent are still projected to increase their market share despite recent increases in crude oil extraction [191]. The present scale of Braskem annual ethylene production is 200,000 t/year (less than 0.2% of total global demand) [39]. The removal of certain market barriers impacting the biofuels sector (e.g., ethanol import duty) would assist growth [180]. In the short term green premiums such as that applied by Braskem to their bio-based polyethylene can work, but in the longer term technological improvements (lignocellulosic feedstocks, economy of scale) must be realised. This is especially pertinent for bio-based solvents, which do not enjoy the luxury of attracting green premiums in the same way consumer products made of plastics do. The massive market size of ethylene means alternative feedstocks will also need to be developed, perhaps avoiding the intermediacy of ethanol as demand on virgin biomass resources intensifies. The volume of present day bio-ethanol transportation fuel use could only satisfy a quarter of polyethylene demand if the feedstock was completely diverted to ethylene production [180]. Therefore more needs to be done to establish secure and sustainable biomass feedstocks for bio-ethanol production, in much greater quantities without causing a negative impact on the environment. Although beyond the scope of this review we can speculate that this could only be in the form of second and (increasingly) third generation biofuels, eliminating competition for arable land.

**Figure 12:**
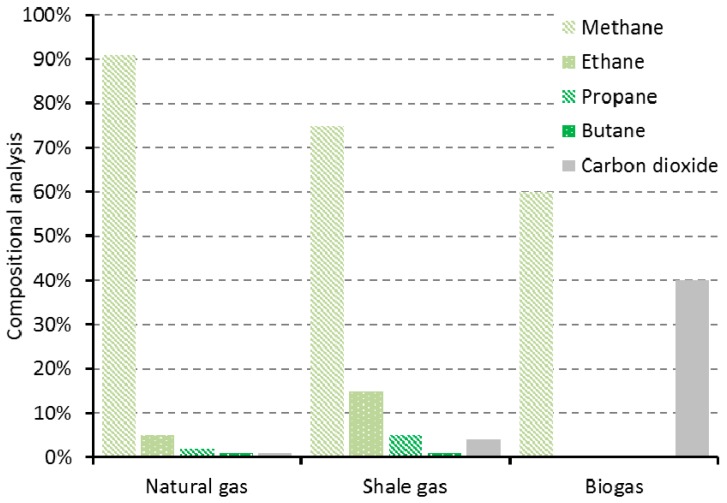
The composition of methane rich gases [117,198].

Another general threat to the bio-based economy that has implications for bio-ethylene as well as bio-gas is shale gas [192,193,194]. Often shale gas reserves are described as a wet gas, meaning it contains higher alkanes (ethane, propane, butane) in greater proportions than typical of natural gas generally [195]. Biogas does not contain higher hydrocarbons and so the interest in wet shale gas exploitation cannot be considered as complimentary to the development of a renewable biogas infrastructure (Figure 12). As a cheap source of hydrocarbons, dehydrogenation of shale gas surplus ethane has resulted in the cheapest present day source of ethylene [196], which consequently influences the price of downstream products such as polyethylene and ethylene glycol (Figure 11). Bio-based PET is made from the ethylene glycol produced by bio-ethylene oxidation, and so this bioplastic is put under a greater economic pressure by shale gas products than it is by present day crude oil prices. Despite the volatility of the chemical feedstock market, bio-based PET products such as PlantBottle continue to be successful because the motivation is there to meet customer expectations regarding corporate and commodity environmentalism [197].

Although it is not necessarily competitive on economic grounds with shale gas, biogas methane could also have a role in the production of the ethylene base chemical, albeit using a different approach. “Methanol-to-olefins” technology can convert methanol by dehydration to ethylene and higher olefins [170]. Usually the ethylene is oligomerised into gasoline, which can be performed as a single integrated process, but other applications of the ethylene are equally feasible.

**Figure 13:**
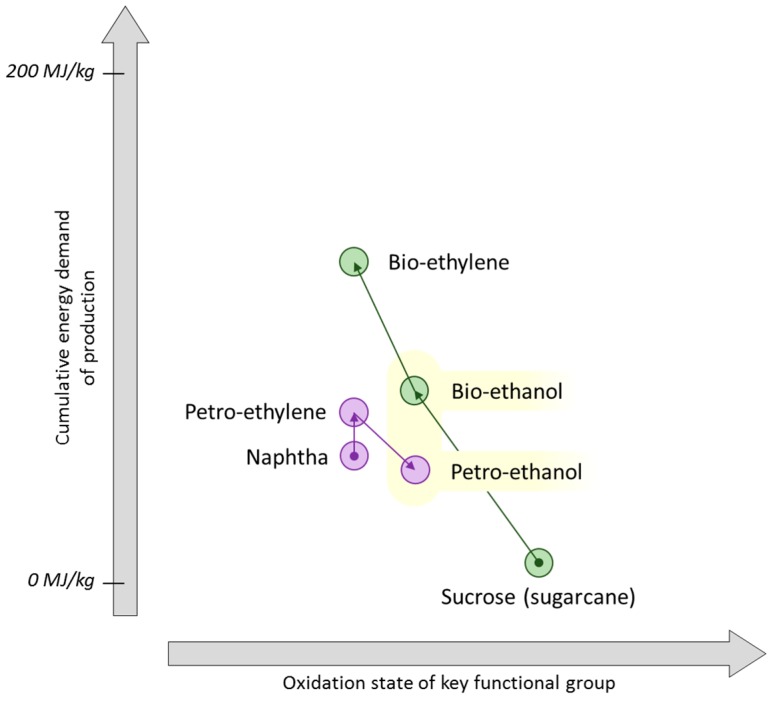
The energy comparison between the manufacture of petrochemical ethanol and bio-ethylene made from sugarcane.

To a certain extent the economic argument for bio-ethylene reflects the cumulative energy demand of production. Because obtaining ethylene from fossil resources requires a similar amount of energy to that required to manufacture ethanol by fermentation of sugars, the downstream bio-ethylene will require significantly more energy to produce than petro-ethylene (Figure 13) [54,136,199]. Note that in more sophisticated analyses the renewable and non-renewable energy input can be compared. This is most evident for the energy embodied in the feedstock. In terms of renewable cumulative energy demand the conclusions reflect much more favourably on the bio-based product than they do here [200]. Note the Figure 13 is drawn on a mass basis, so that the hydration of fossil derived ethylene (28 g/moL) to ethanol (46 g/mol) causes a reduction in CED when valued in units of MJ/kg due to the low CED of water.

**Scheme 3:**
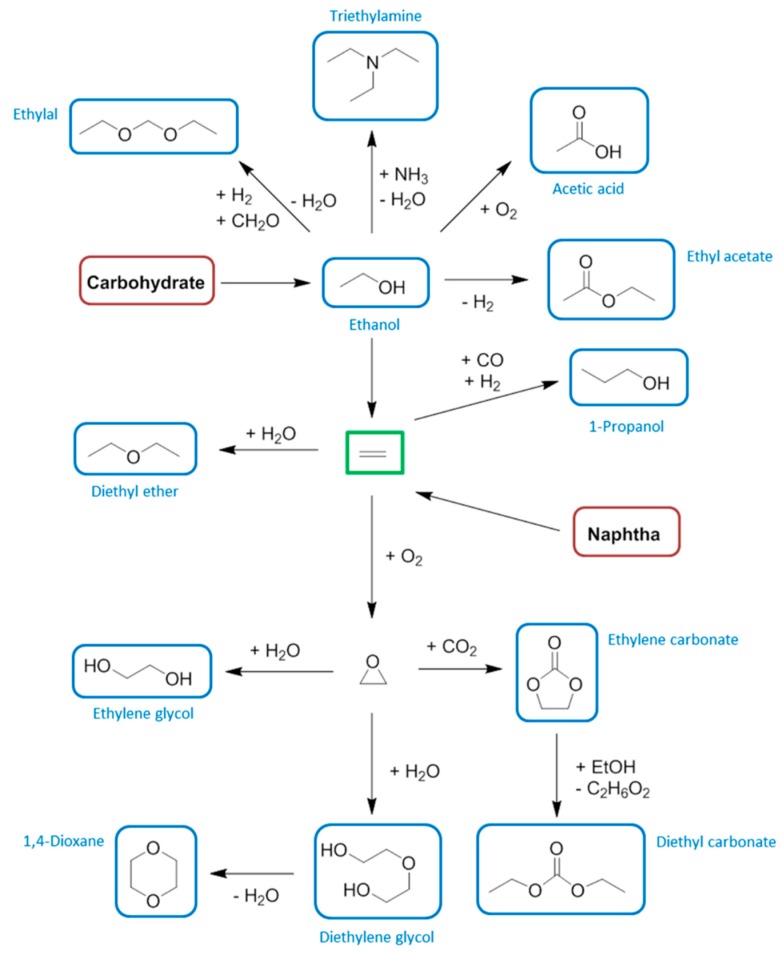
The ethylene synthetic platform relevant to solvent production. Key: red = feedstocks, green = key intermediate, blue = solvents.

Despite the multiple options available, it should be reiterated that at present the only significant route to bio-ethylene is the dehydration of bio-ethanol. It may also be possible to generate industrially relevant volumes of ethylene in the future using alternative processes such as direct biological engineering approaches [201]. Bio-ethylene is currently used to make PE, and PET via ethylene glycol. This ethylene glycol intermediate provides the only currently mass-produced bio-based solvent from the bio-ethylene downstream production chain. Ethylene glycol is also of academic interest to produce methanol [202,203]. However, more generally, ethylene leads to the solvents 1-propanol, diethyl ether, and chlorinated solvents such as 1,2-dichloroethane (DCE) amongst others (Scheme 3). Following the precedent of the petrochemical industry, ethylene oxide is then used to produce 1,4-dioxane, ethylene glycol and related glycol ethers, and a variety of carbonate solvents. Whereas many ethers are not considered green for health or stability (specifically peroxide forming) reasons, carbonate solvents, both cyclic and acyclic, are part of the growing catalogue of greener organic solvents, presenting environmental benefits as well as reasonable health and safety profiles compared to similar solvents [34,141,204].

**Scheme 4:**
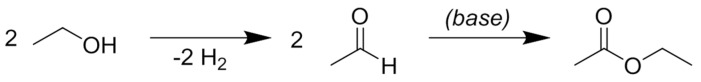
Production of ethyl acetate is possible using the Tishchenko reaction of acetaldehyde formed by the oxidation of ethanol.

In comparison to the largely hypothetical routes to bio-based solvents from ethylene and ethylene oxide, bio-ethanol is more widely used as the basis of solvents actually available on the market (Scheme 3). Conversion to acetic acid by an oxidative fermentation has already been discussed. Ethylamines are produced by the multi-national chemical manufacturer BASF (Badische Anilin- und Soda-Fabrik) by the reaction of bio-ethanol with ammonia [205]. Ethyl acetate is produced renewably from ethanol by the Swedish company Svensk Etanolkemi AB, known as SEKAB [59], and by an equivalent process performed as a Davy-Sasol joint venture (Scheme 4) [58]. Ethylal made from bio-ethanol is available [206], as are ethyl esters such as the neoteric solvent ethyl lactate [68].

Ethylene is the most useful of the olefin base chemicals in terms of its potential to produce a large range of solvents. This opportunely mirrors the greater production of ethylene compared to other olefins globally [39]. Together, bio-ethanol and bio-ethylene incorporated into the base chemical sector can provide a great deal of flexibility regarding the production of solvents. In combination with syngas the majority of conventional oxygenated solvents for most purposes could be created from renewable sources of bio-ethanol. This brief analysis would suggest many bio-based solvents will not rely so heavily on the conventional base chemical ethylene, with a significant portion by-passing ethylene and instead produced directly from bio-ethanol. The ways in which bio-ethanol can also be integrated into the production chains of solvents traditionally synthesised from propylene and butene is discussed subsequently. Furthermore many neoteric bio-based solvents could incorporate bio-ethanol as the esters of other intermediates (ethyl lactate, diethyl succinate, *etc*.).

## 7. Propylene

Propylene is another key base chemical, presently required for the production of solvents such as acetone, methyl isobutyl ketone (MIBK), isopropanol, 1-butanol, and their respective esters from fossil resources. Much the opposite state-of-the-art describes bio-propylene compared to bio-ethylene. Although interest is high with a number of candidate molecules available, the conversion of biomass into propylene is less well developed than the production of bio-ethylene (which by contrast is obtained through one source). Fermentation products such as acetone (commercial) [70], or isopropanol (not commercial) [207], are not ideal intermediates for bio-propylene production, themselves presently formed chiefly from the oxidation of petrochemical propylene. This often unnecessary reversal of the production chain is likely to introduce the familiar economic barriers on top of the evident technology limitations. Despite this the concept of a C_3_ biorefinery for propylene manufacturing is being developed, driven by the strong desire to create bio-based olefins regardless of the challenges [208].

Regardless of the hurdles, interest in the manufacturing of products from bio-propylene (once again driven by the bioplastic industries) is sufficient enough to have secured some progress towards this goal. Pilot plant scale developments exploring a variety of different options suggest the technology will soon reach fruition and yield commercial products. Promising feedstocks include glucose in a direct fermentation to propylene [209]. The French company Global Bioenergies also claim to produce a number of other gaseous fermentation products, including isobutene (2-methylpropene) [210]. This advance eliminates the energy intensive separation usually required to obtain solid or liquid chemicals from dilute fermentation broths. We have seen with the cumulative energy demand of acetic acid (Figure 10) and ethanol (Figure 13) production that fermentations are a challenge to operate with an energy efficiency comparable to petrochemical industry processes.

Fermentation to give olefins directly will probably be a minor contributor at first to the bio-propylene market and its downstream products [211], with more conventional cracking and isomerisation reactions an obvious alternative. These less adventurous approaches to bio-propylene production include the use of bio-ethylene from ethanol dehydration. Metathesis of dimerised ethylene (isomerised to 2-butene) with further ethylene will create propylene [212,213,214,215]. Bio-butanol could also be used in this set-up to provide a source of butene. The impetus for this work is the opportunity to create bio-based polypropylene [216,217,218], although enthusiasm to realise this as a commercial enterprise has lessened of late [219]. In attempts to streamline the conversion of ethanol to propylene, academic work has shown the direct conversion of ethanol to propylene is possible with certain heterogeneous catalysts [220,221,222,223]. The three most active catalysts (nickel ion-loaded mesoporous silica, scandium/indium(III) oxide, and yttrium supported on ceria) each have different mechanisms of catalysing the reaction [221]. Deriving propylene from bio-ethanol is a helpful approach to ensure availability and the price competitiveness of the feedstock. However this places production at the mercy of the liquid biofuel market. Methanol-to-olefins technology is relevant for the possible synthesis of propylene from bio-based methanol, with multiple options open in terms of processing technologies [169]. Selectivity to propylene can be tuned through catalyst (e.g., zeolite) development [224].

Additional procedures relevant for the production of bio-propylene utilise vegetable oils. Neste Oil will soon begin supply of bio-based propane, a by-product of their vegetable oil valorising bio-based gasoline process [225,226]. The availability of propane in certain geographical areas has encouraged producers to dehydrogenate this alkane to yield propylene, and so a precedent for this procedure is already established [227]. The economic competitiveness of this product (compared to shale gas propane as an example) is not yet clear. Alternatively vegetable oils can be subjected to steam cracking to give the different olefin streams. Propylene yields of 20 wt % have been reported [228]. A summary is tabulated below (Table 2).

**Table 2:** Potential sources of bio-based propylene for the synthesis of solvents.

Feedstock	Conversion Technology	Readiness Level	Reference
Vegetable oil	Vegetable oil steam cracking	Commercial	[225,226]
Glucose	Direct fermentation	Pilot plant	[209]
Methanol	Methanol-to-olefins	Commercial with fossil derived feedstock	[229]
Ethanol	Metathesis	Pilot plant	[214,215]
	Direct conversion	Not commercial	[221]
Butanol	Metathesis	Not commercial	[230]

The reactions just summarised bear a resemblance to conventional petrochemical propylene processes. Although the classic view of propylene production is naphtha cracking, the low yield has meant more specific, targeted syntheses of propylene make important contributions within the existing chemical industry, such as the aforementioned conversions of propane, ethylene and methanol-to-olefins technology. The diversity seen across propylene producing facilities probably helps the adoption of bio-based feedstocks at appropriate scales for the maturity of the technology, because the biorefinery is not competing with a single dominate petrochemical process that uses a feedstock alien to biomass resources.

**Scheme 5:**
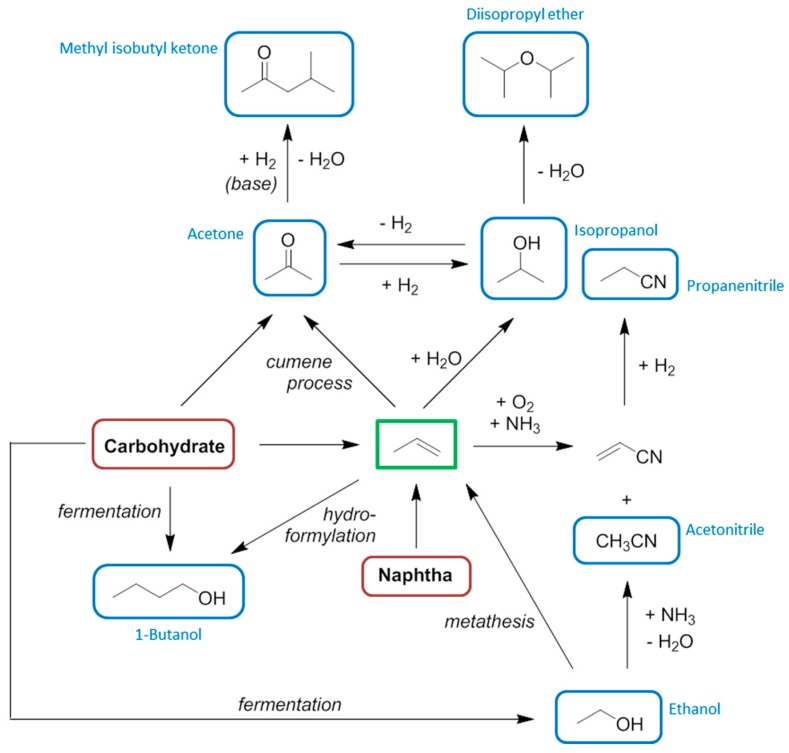
Propylene solvent synthesis tree. Key: red = feedstocks, green = key intermediate, blue = solvents.

**Scheme 6:**
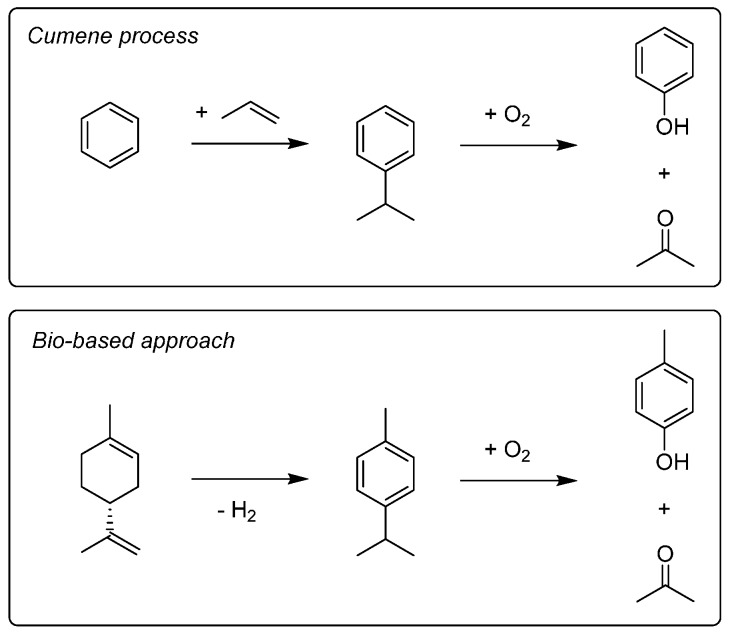
A comparison between the cumene process and the terpene (limonene) equivalent to give acetone as a by-product.

The solvents presently produced from propylene are given in the following diagram (Scheme 5). Not included are esters of the alcoholic solvents, or the diol solvents, which are covered later. It is quite probable that in the future a lot of the solvents shown in Scheme 5 will not be produced from bio-propylene even if it should become available at an appropriate price from sustainable resources. Firstly diisopropyl ether is a dangerous peroxide-forming agent and should no longer be used as a solvent. Methyl isobutyl ketone (MIBK) is a suspected carcinogen [231]. 1-Butanol is a fermentation product and does not require a propylene intermediate [69]. Acetone is also fermentation product co-produced alongside 1-butanol, and could be transformed into bio-based isopropanol as required. Propanols can also be produced by the hydrogenolysis of glycerol when tuned to avoid the usual diol products [232]. Acetone could also be acquired from the processing of the essential oils of citrus fruit, although the route is long for the manufacture of a low cost commodity compound. It would be required that limonene is dehydrogenated to *p*-cymene [233], which in turn can be oxidatively cleaved to give acetone and *p*-cresol as per the analogous petrochemical cumene process (Scheme 6). It is worth noting that isopropanol was at one time converted to acetone [234], but now when the cumene process generates a market surplus of acetone, the reverse procedure is put into practice [235].

Based on this assessment it would seem that bio-based propylene is not vital for the future of the solvent manufacturing industry. Nevertheless the large plastics market is enough of a pull to ensure bio-propylene comes on-stream as a monomer within the next few years [214]. Solvent suppliers would be certain to adopt a renewable intermediate such as bio-propylene should it become price competitive with fossil derived propylene and a regular voluminous supply established (ideally from a choice of suppliers).

However, one important solvent that specifically requires propylene (not acetone, propanol, *etc*.) for its synthesis is acetonitrile. Acetonitrile is a major solvent because of its use in chromatography [236], and it is co-produced with acrylonitrile from propylene [237]. Unfortunately this means the availability and price of acetonitrile is strongly related to the acrylonitrile plastics market, and a shortage even occurred in 2008 [238,239]. The acrylonitrile could alternatively be hydrogenated to give propanenitrile as a complimentary alternative to acetonitrile. It is possible to make acetonitrile from ethanol [240], but not proven on an industrially relevant scale (especially with regards to establishing dedicated production for a product that can be obtained cheaply as a by-product). Nevertheless it would fit with the general observation that an ethanol-derived basis of chemical production could mostly supplant the role of the base chemical propylene where bio-based solvents are concerned.

**Figure 14:**
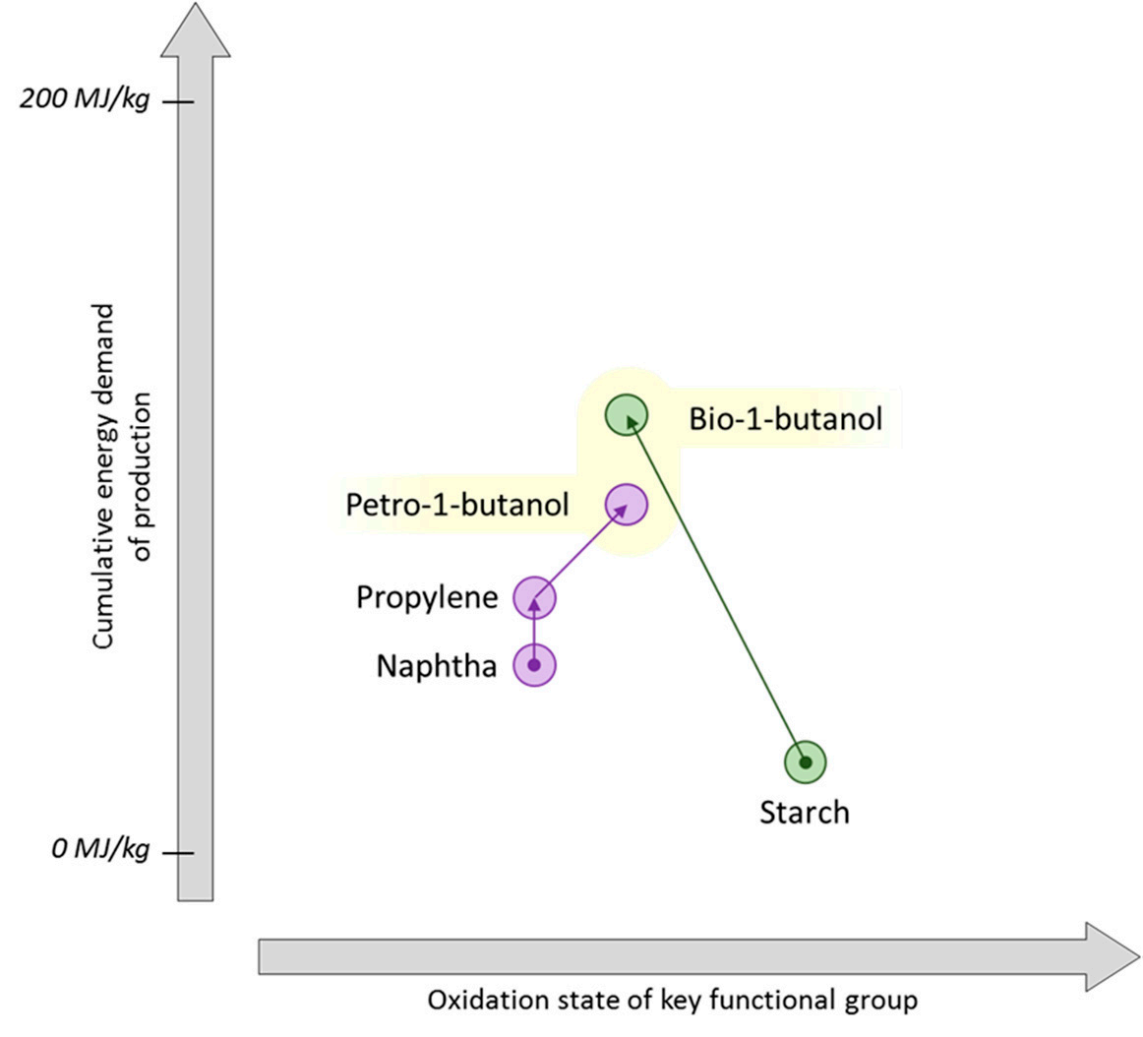
The cumulative energy demand of 1-butanol production.

The contrast between the traditional propylene synthesis tree and viable biorefinery approaches can be further investigated with the case study of 1-butanol. Conventionally the hydroformylation of propylene is applied to yield 1-butanol via butanal [241,242]. Processes that can provide the bio-based propylene intermediate are redundant here because of the competing fermentation route directly to the alcohol. Bio-1-butanol is mainly considered as a fuel [243]. The energy required to produce bio-butanol by fermentation of carbohydrates is similar to petrochemical 1-butanol made via propylene (Figure 14) [136,137,244]. This bodes well for the transition of this multi-million tonne market to a renewable feedstock [245]. It is reasonable to assume given the present state-of-the-art that a process to produce bio-propylene would not be less energy intensive than the fermentation indicated in Figure 14.

Of the less orthodox feedstocks that can be utilised for the synthesis of solvents typically derived from propylene, glycerol offers one of the most promising options [246]. The low price of glycerol, now abundant as the by-product of bio-diesel production, makes it an obvious target for hydrodeoxygenation to bio-propylene [247]. However reviews on the chemistry of glycerol do not focus on the synthesis of propylene [248,249,250], suggesting it is not a viable reaction (economic rationale may trump technological reasons). Propylene glycol is the typical end product of the type of reduction chemistry usually applied to glycerol [251], and as a lower value product further conversion to propylene is not necessarily appealing. The value chain reflects the fact that propylene glycol is presently made from propylene, and so in terms of the synthesis of bio-based 1,2-propanediol, there is a clear benefit to it occurring directly from the glycerol substrate anyway. In terms of offering an easily diversifiable platform molecule this chemistry is limiting but presumably more financially rewarding. A complimentary fermentation process can convert glycerol to the niche solvent 1,3-propanediol, which has been modelled with the conclusion of high techno-economic potential [252]. Note that in reality DuPont-Tate & Lyle manufacture bio-based 1,3-propanediol by means of a fermentation of corn starch and not glycerol [253].

**Scheme 7:**
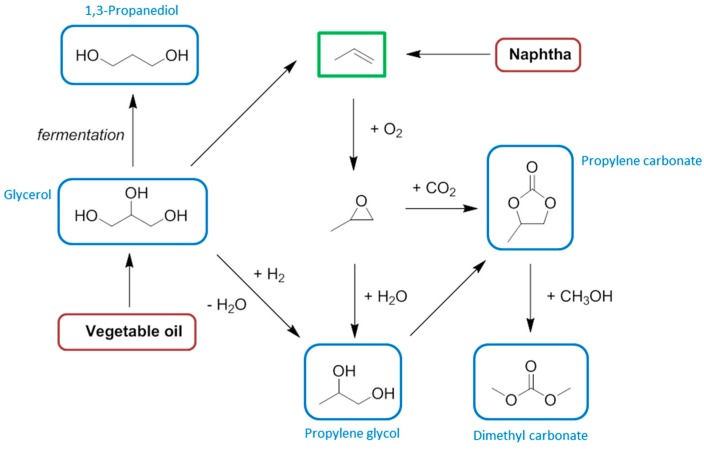
Some solvent derivatives of propylene via glycerol and propylene oxide. Key: red = feedstocks, green = key intermediate, blue = solvents.

A representation of glycerol derived solvent products and their relation to the propylene synthesis tree is given in the following diagram (Scheme 7). The shortcut to 1,2-propylene glycol is quite obvious, avoiding propylene oxide as an intermediate. Because bio-based propylene does not appear to be crucial for the synthesis of bio-based solvents, solvents currently derived from propylene oxide may be prevented from becoming significant additions to the bio-based solvent catalogue. This is not the case for bio-ethylene, where bio-based ethylene oxide is already used to produce ethylene glycol. The solvent for which a lack of propylene oxide is most important is propylene carbonate. Cyclic carbonates produced from the reaction between epoxides and carbon dioxide are used as solvents in lithium ion batteries [254,255]. Thankfully the direct use of the diol to form the analogous carbonate is well-matched to the current status of bio-based chemical market and justly generating interest [256,257].

**Figure 15:**
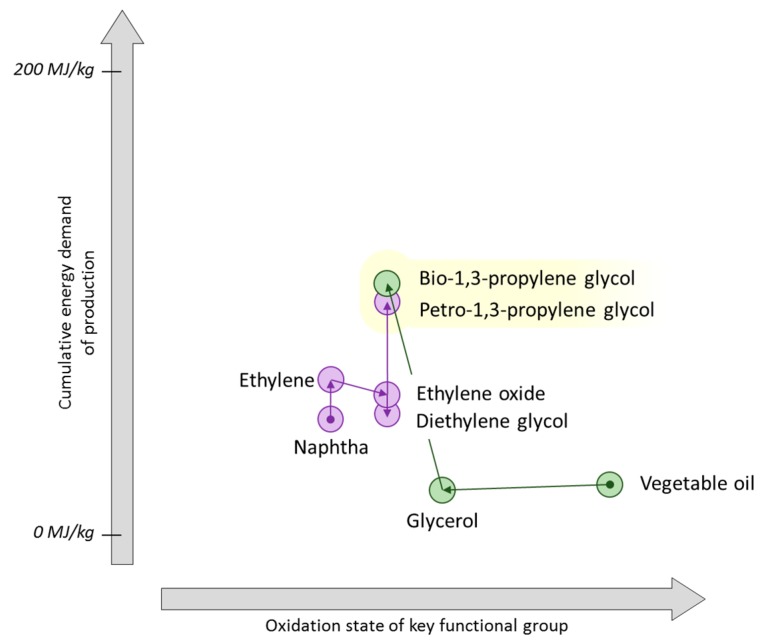
The cumulative energy demand of glycol solvent production.

Considering the market opportunities for glycerol-derived diols, ethylene glycol is presently the most ubiquitous petrochemical equivalent found in these roles. It is used as an antifreeze and for heat transfer applications [258]. Substitution with the less toxic propylene glycol is clearly beneficial [259], but in reality prevented by the higher price of this alternative [12]. The use of a biomass feedstock is not the chief issue from a technological perspective, for fossil-derived 1,3-propanediol (made by the hydroformylation of ethlyene glycol) and its bio-based equivalent (made by fermentation) require a similar amount to energy to produce (Figure 15) [54,136,260]. The latter has been commercialised [261]. Instead it is the low cost of ethylene glycol ethers that is creating a barrier to change, represented in the following CED diagram by diethylene glycol (data for ethylene glycol was not available) [136]. Note the value for the CED of glycerol production given in the literature is for the entire vegetable oil feedstock production and biodiesel manufacturing process (166 MJ per kilogram of glycerol) [251]. In this work this value is distributed across both the bio-diesel and glycerol products on a mass basis, reducing the CED of glycerol production to a more intuitive 19 MJ/kg. Replacement of ethylene glycol with glycerol derived C_3_ diols may become more widespread as the cost of production is reduced with expanding operational scale, or perhaps it will be helped by regulation relating to the use of ethylene glycol (particularly in applications where contact with drinking water or food is concerned) [262].

Rather than progressing to conventional solvents via glycerol, a great deal of research into bio-based solvents concerns novel glycerol derivatives and deep eutectic mixtures [263]. The viscosity of glycerol itself can be a limitation to its application as a solvent at room temperature [264], and so glycerol formal [265], and related solvents are produced in order to valorise the cheap glycerol resource [266]. The plasticiser triacetin is another glycerol-derived product of interest [267]. Although it is not the focus of this review, it is important to consider how neoteric bio-based solvents may influence the conventional solvent market. If this causes a conventional solvent to fall out of favour, securing a biomass feedstock and establishing a synthesis is not warranted.

Along with glycerol, lactic acid is the other C_3_ bio-based chemical realising its potential on a necessary scale to generate interest in the area of bio-based solvents. Whereas glycerol is a by-product of the biofuel industry, lactic acid is manufactured primarily for poly(lactic acid) (PLA) synthesis [268], but in doing so also provides an opportunity for neoteric bio-based solvents. Lactic acid has been demonstrated to be a solvent applicable to chemical synthesis [269]. The ethyl ester is marketed as a solvent, especially as part of cleaning formulations [270,271,272]. The production and characteristics of ethyl lactate have been reviewed separately [273]. In terms of conventional solvents, lactic acid is also a possible feedstock for propylene glycol production [274,275].

With this overview of alternative feedstocks for propylene derived solvents complete, the present day situation would seem neither advanced nor well defined. Instead of bio-propylene being used as the obvious drop-in replacement, glycerol and bio-ethanol in particular will continue to assume importance as intermediates, with lactic acid also providing a useful C_3_ molecule to compliment the development of bio-based solvents. Bio-1-butanol production by fermentation offers a way to bypass the need for propylene, as do several other transformations of biomass covered in the preceding text. This approach suggests an increase in more specific chemical manufacturing processes is needed to supplement the functionalisation of base chemicals such as propylene.

## 8. Butenes and Butadiene

**Scheme 8:**
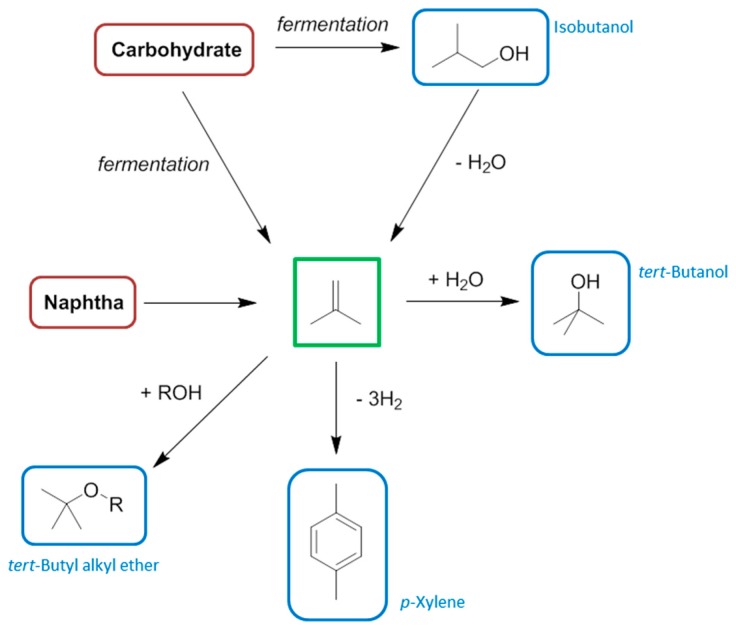
Hydrocarbons and related oxygenates obtained through fermentation to isobutene. Key: red = feedstocks, green = key intermediate, blue = solvents.

The different C_4_ olefins (1-butene, 2-butene, isobutene, and butadiene) all have a role in the present day chemical industry. Their impact in terms of downstream solvent products does not compare to the lower olefins, with only a few important solvents produced from butenes. The most important bio-based commodity chemicals containing four carbon atoms are not generally compatible with the butene synthesis tree, because a chemical such as 1-butanol for example, now produced by fermentation, is actually most commonly derived from propylene using hydroformylation chemistry as previously discussed. Succinic acid is another primary fermentation product, and this is actually a downstream intermediate from benzene within the context of the petrochemical industry. The most established bio-based chemical compatible with the present day butene base chemical infrastructure is actually isobutanol (2-methylpropanol) [276]. The isobutanol can be dehydrated to give isobutene (Scheme 8) [277]. On a smaller pilot scale a direct fermentation to the gaseous olefin has also been demonstrated [278], including from waste biomass [279].

The main driver for producing bio-based isobutene is so that *p*-xylene can be manufactured as an intermediate for the PET plastic industry (Figure 1). This presents an opportunity for bio-based isobutanol and bio-based *p*-xylene to be siphoned from the bioplastic production chain and be applied as solvents. Although its primary uses are the production of synthetic rubber and fuels [280], isobutene is also used to produce *tert*-butyl alcohol and alkyl *tert*-butyl ethers. Although methyl *tert*-butyl ether (MTBE) is under scrutiny because of the contamination of groundwater it has caused in its role as a fuel additive [281,282], its ethyl analogue is produced from bio-ethanol to provide an alternative [283]. Isobutene is also used to create *tert*-butyl esters, of which *t*-butyl acetate is recognised by the pharmaceutical company GlaxoSmithKline (GSK) as the greenest of the commonly available ester solvents within their solvent selection guide [34].

**Scheme 9:**
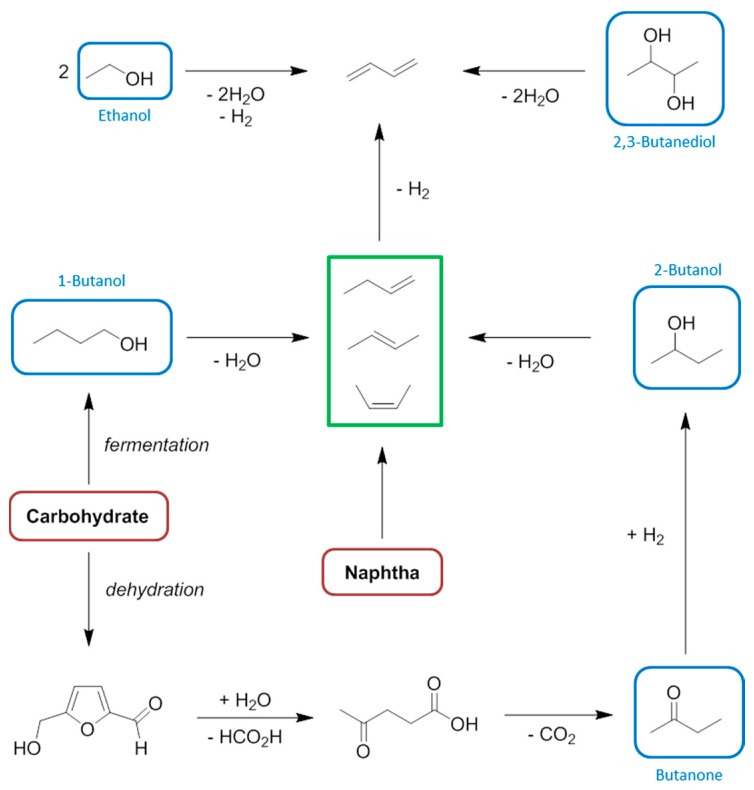
Routes to butenes and 1,3-butadiene from bio-based alcohols. Key: red = feedstocks, green = key intermediate, blue = solvents.

To generate bio-based 1-butene and 2-butene, dehydration of 1-butanol could be applied. In the dehydration of an alcohol, a secondary alcohol is preferable and so downstream processing of bio-based 1-butanol could feasibly be overshadowed by an alternative feedstock. However fermentation to provide 2-butanol is less well developed as a technology compared to 1-butanol production [284]. One alternative approach in order to obtain 2-butanol is to utilise levulinic acid as an intermediate. Production of levulinic acid by the acid-catalysed thermal treatment of carbohydrate is poised to gain a significant share of the bio-based chemical market [285]. Decarboxylation gives butanone, which can be reduced to 2-butanol and then potentially give a butene [51] (Scheme 9). This route is probably too long to provide an economically attractive base chemical and not only from the perspective of the energy expenditure required to remove the chemical functionality. The synthesis progresses through potential solvent molecules *en route* to butene, therefore this source of butene becomes redundant for future solvent production. Butanone is known to have undesirable effects on human health and the environment, prompting the development of alternative solvents [286].

Ethanol can once again be viewed as a candidate precursor for bio-based platform molecules, this time reduced and coupled to give 1,3-butadiene (Scheme 9) [287,288,289]. Butadiene was historically produced from ethanol [12]. Global Bioenergies has patented a process for producing 1,3-butadiene from alkenols via enzymatic dehydration [290]. Dehydration of 2,3-butanediol, obtainable from renewable resources [291], is also an option under investigation [292]. Regardless of the potential biomass feedstock, 1,3-butadiene does not presently have a role in the production of solvents, for the obvious diol products are obtained by fermentation directly. Uses of 1,3-butadiene are the production of synthetic rubber and its copolymers such as acrylonitrile-butadiene-styrene (ABS) [293]. It is however possible to use 1,3-butadiene in the synthesis of cycloalkenes by means of the Diels-Alder reaction, and therefore cycloalkanes can in theory be accessed. In that lies potential for bio-based hydrocarbon solvents but alternative routes by hydrogenation of bio-based aromatic compounds and terpenes may be more realistic.

Less specific technologies are also geared towards producing renewable butenes and butadiene, such as the Fischer-Tropsch process and aqueous phase reforming using sugar substrates [294,295]. These chemicals will find their way into the general stock of intermediates as well as being reserved for higher value bio-based production streams. The same is also true of the other hydrocarbon base chemicals as previously discussed, and processes are now being investigated where a mixed feed of biomass and a conventional fossil feedstock is used to create olefins of variable bio-based content [296,297,298]. However the greatest ingenuity has arguably been reserved for the challenge of developing processes to deliver renewable aromatic hydrocarbons, which will now be discussed.

## 9. Aromatic Base Chemicals

A variety of different aromatic solvents once enjoyed widespread use as solvents, but just as benzene was superseded by toluene, users of toluene are now under pressure to change to another solvent [299]. This is an important problem to address because of the large demand for toluene and the other aromatic solvents globally [300,301]. The most likely candidates to be applied as immediate substitutes are the xylenes, with the majority of industries seeking to keep ahead of the legislative control of chemicals without investing in long-term substitutes. The use of the aromatic base chemicals as solvents is objectionable, all being on the Hazardous Air Pollutants (HAP) list of the EPA [302], and several other prominent lists of chemicals requiring substitution (Table 3) [303]. Ideally substitution of undesirable aromatic solvents would be conducted in such a way as to completely sidestep the advancing regulation and legislative restrictions, but short-term options will continue to be used when commercial operations operate on the basis of annual economic performance, not long-term objectives addressing sustainability without obvious and immediate profitability [304]. The commercial alternatives to aromatic solvents tend to be specially formulated blends of oxygenated solvents [300], with n-propyl propanoate also promoted as a non-HAP alternative to toluene in specific applications [305].

**Table 3:** Issues surrounding aromatic solvents, as gauged from safety data sheets (some hazards may be unknown).

Solvent	Safety Concerns	Acute Toxicity	Chronic Toxicity	Environmental Issues
Benzene	Flammable	Irritant and toxic	Carcinogen and mutagen	HAP
Toluene	Flammable	Irritant and toxic	Suspected reproductive toxin	HAP
*p*-Xylene	Flammable	Irritant	Not known	HAP
*p*-Cymene	Flammable	Irritant	Not known	Not known
Ethylbenzene	Flammable	Irritant and toxic	Possible carcinogen	Harmful to aquatic life

**Scheme 10:**
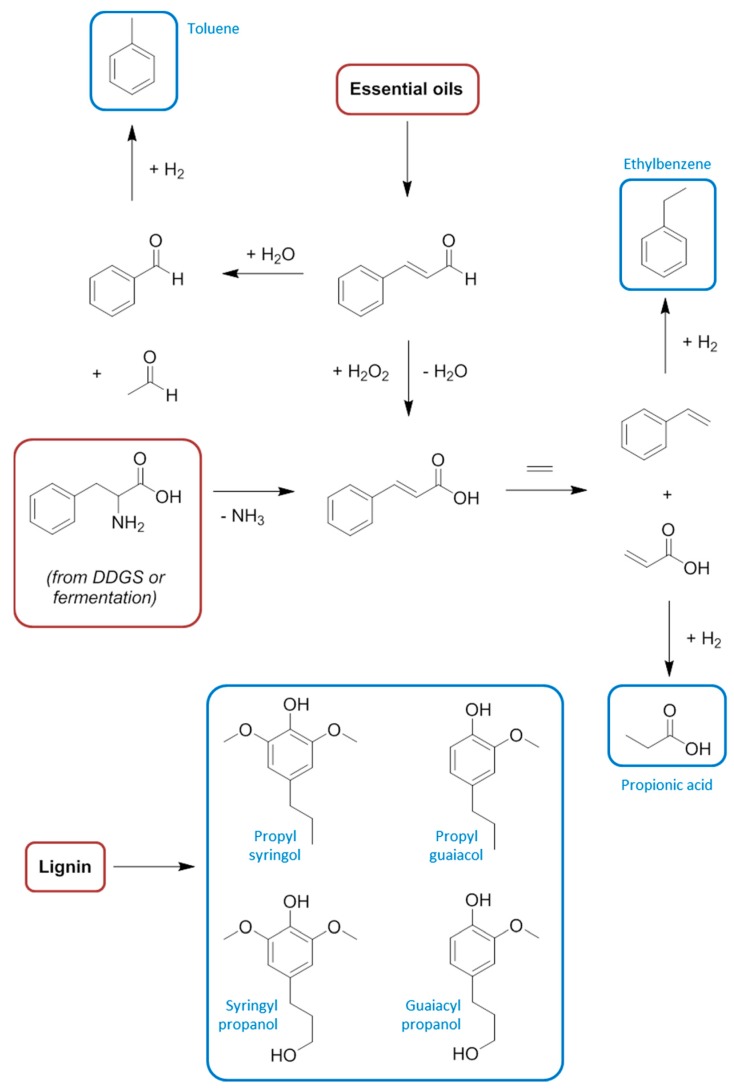
Possible methods for the synthesis of some bio-based aromatic solvents. Key: red = feedstocks, blue = solvents.

As might be expected, the driver for producing renewable aromatic compounds is not for low value solvent uses but for the larger fuel market and higher value chemical intermediate roles. Chemically, the obvious biomass resource from which to make aromatic solvents would be lignin, or alternatively essential oils [306,307], select amino acids [308], and any other compounds already containing aromatic functionalities (Scheme 10). Unfortunately lignin has proven resistant to most attempts to retrieve useful aromatic compounds from it at competitive prices, vanillin a notable and valuable exception [309]. Nevertheless neoteric aromatic solvents made from lignin have been proposed [310]. These solvents are produced in an orthogonal manner to the BTX base chemicals, with cinnamaldehyde or cinnamic acid often a useful intermediate.

Also bypassing the petrochemical base chemicals, limonene has received attention as a precursor to the aromatic solvent *p*-cymene (Scheme 6). Both molecules have been used as solvents in different applications [311,312,313], but the synthesis of renewable *p*-cymene is limited to academic discussions and theoretical assessments at present [233,314,315]. *p*-Xylene can also be made from limonene [316,317], but this is not favourable compared to the isobutanol route covered in the previous section (Scheme 8).

**Scheme 11:**
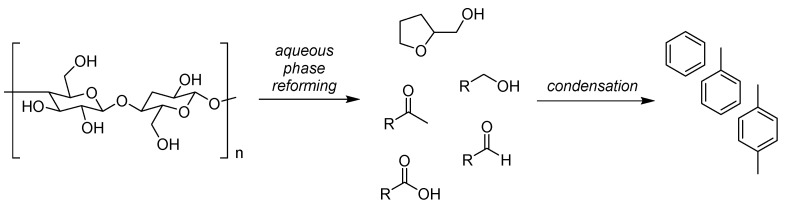
An aqueous reforming process applied to carbohydrate as a precursor to aromatic solvent production.

Catalytic pyrolysis of cellulose provides the most promising option for the large-scale manufacture of bio-based aromatics, tuned to give benzene, toluene and xylenes [318,319]. Glycerol is an alternative feedstock [320]. A pilot plant has been designed to demonstrate the manufacture of these bio-based aromatic compounds, although the anticipated price and scale will at first forbid any significant solvent applications [321]. The process requires zeolite catalysts and a fluidised bed reactor to produce a BTX product stream from wood and other sources of carbohydrate [322,323]. A similar but two stage process commercialised by Virent transforms carbohydrates by aqueous reforming into intermediates that can then be reduced to liquid aromatic compounds (Scheme 11) [324,325]. Recently *p*-xylene made using this technology has been used to create 100% bio-based PET for the first time [326,327].

As the use of aromatic solvents is phased out to only crucial roles, the technologies reviewed above will probably not serve as sources of future solvents, Instead the primary market will be as intermediates for plastics production (e.g., PET and polyurethanes). Some applications will conceivably be reliant on aromatic solvents because there is no presently available option to move to another solvent type. These processes will continue to operate unchanged unless more inventive solutions are developed as aromatic solvent substitutes. This is certainly one purpose of the neoteric solvents, which are now reviewed in brief to finalise this perspective on the synthesis of bio-based solvents.

## 10. Neoteric Bio-Based Solvents

It is important to remember that only so much progress can be made towards a more sustainable and greener chemical industry by continuing to produce the same substances, just sourced from renewable resources. The toxicity and environmental impact of many common solvents means they should not be used, and cannot be used if restricted by regulations. Fittingly, most of the academic interest in green solvents is concerned with neoteric solvent systems, but often petrochemical in origin. Ionic liquids are the most widely studied [328]. Studies on supercritical fluids, usually carbon dioxide [329], account for far less publications but it is more common in industrial applications [124]. Water is the other important green solvent in terms of the quantity of research published [330,331].

Although the aforementioned solvents are interesting, particularly from the academic perspective, neoteric solvents are not significantly changing the chemical industry by solely relying on their unique properties to generate uptake. That said, the need for new, perhaps unconventional solvents is important because of the imminent impact of REACH and related legislation. When conventional solvents are taken out of common use, an opportunity, even a necessity arises for new solvents with diminished environmental, health and safety concerns to be introduced.

**Scheme 12:**
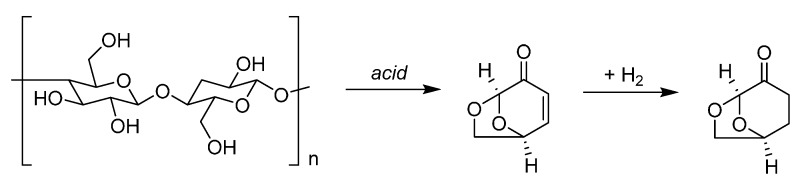
The synthesis of Cyrene from cellulose via levoglucosenone.

A balance between retaining the familiarity of using conventional organic solvents (along with the established chemical industry infrastructure) but attaining sustainability in solvent use may best be achieved using neoteric bio-based solvents. This is not to say that the solvents reviewed in this article will shortly become redundant; bio-ethanol, acetone, ethyl acetate and the like will surely be used as solvents into the future indefinitely. What is evident is that different types of solvent are also required, and they will need to be structurally dissimilar to their predecessors to avoid the same environmental, health and safety issues. Not only their chemical structure be will unconventional. The feedstock and intermediates will differ from the petrochemical base chemicals. It is this broader chemical intermediate basis provided by biomass that creates the differences in the final chemical functionality of bio-based products compared to petrochemicals. A recent example is the bio-based solvent dihydrolevoglucosenone (Cyrene), recently identified as a replacement for some applications of NMP [40]. It is produced from levoglucosenone, a bicyclic, multifunctional chemical intermediate with two chiral centres. Levoglucosenone is not at all similar to the petrochemical base chemicals, except that it is produced from a (cellulose) feedstock in one chemical step (Scheme 12).

**Scheme 13:**
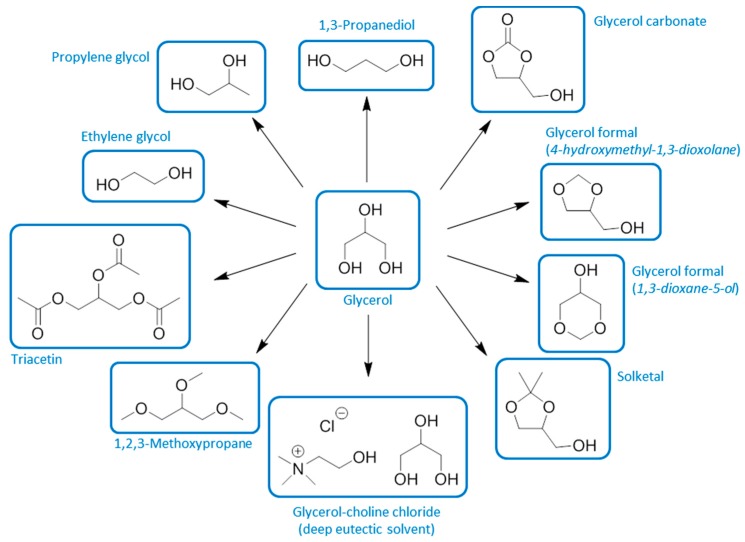
Solvents produced from glycerol [71,266,333].

Neoteric bio-based solvents are typically high boiling, and can be more viscose than conventional solvents, which can present challenges when introduced to the long established reactor chemistry and distillation recovery infrastructure. The obvious example is glycerol, which because of its abundance and low value as a by-product of bio-diesel manufacturing has also led to the development of several other neoteric solvents made from it (Scheme 13). Nevertheless glycerol itself has also been investigated as a neoteric solvent [332].

Solvent-specific issues also exist for the other neoteric classes of solvent, with use of water (recovery and contamination issues), ionic liquids (toxicity and recovery issues), and supercritical fluids (incompatible with traditional chemical plants) also met with barriers. Some commercial and pilot plant stage neoteric solvents made from biomass are presented in Table 4 to demonstrate the hurdles are not insurmountable. In fact, a great deal of interest exists in these products, and that has stimulated significant progress in academic circles, which is now trickling into commercialisation [13]. Note that the only entry in Table 4 that is completely synthesised from conventional base chemicals is dimethyl ether. Although this suggests there will be a limited need for the petrochemical intermediates in future solvent manufacturing, the market share of these solvents is small at present compared to toluene, methanol, and other more familiar solvents.

**Table 4:** A selection of neoteric bio-based solvents that are either commercially available or produced in demonstrator plants.

Solvent	Source	Manufacturer
Glycerol	Vegetable oil	Cargill [334]
Solketal	Vegetable oil	Solvay-Rhodia [335]
Glycerol formal	Vegetable oil	Lambiotte & Cie [336]
Limonene	Essential oil	Various [337]
Lactic acid	Fermentation of carbohydrate	Cellulac [68]
Ethyl lactate	Fermentation of carbohydrate	Cellulac [68]
Diethyl succinate	Fermentation of carbohydrate	BioAmber [338]
Fusel oil	Fermentation of carbohydrate	Various [339]
1,3-Propanediol	Fermentation of carbohydrate	Dupont-Tate & Lyle [261]
1,2-Pentanediol	Reduction of carbohydrate	Pennakem Europa SAS [340]
Cyrene	Reduction of carbohydrate	Circa [160]
2-MeTHF	Reduction of carbohydrate	Pennakem Europa SAS [341]
γ-Valerolactone	Reduction of carbohydrate	Various [339]
Dimethyl isosorbide	Reduction of carbohydrate	Roquette [342,343]
Dimethyl ether	Gasification	Chemrec [344]

When avoiding certain chemical functionalities in order to prevent toxicity or particular environmental impacts, completely satisfactory solvent substitutions are difficult to achieve. It will certainly be the case that several solvents will be needed to replace each of toluene, NMP, chloroform, *etc.*, in very application specific ways depending on what is required of the solvent. Hence the diversity of solvents will probably increase with time, and that diversity will rely on several feedstock options and different types of intermediates. One downside is that many neoteric solvents may therefore continue to be mostly confined to niche applications, as they are today. In turn, this means they can only be manufactured on relatively small scales, and that may have implications for the sale price, effectively trapping the neoteric solvents in their respective niches. It was for this reason that we have described a more subtle approach to developing bio-based solvents in a sympathetic manner to existing processes, making familiar products with established markets. Some solvents regarded as neoteric in this work (strictly speaking meaning “new”) already have an appreciable market share because they are not actually new products. We have used neoteric only to imply the structure of the solvents is not similar to petrochemical products. Terpenes are one such type of established ‘neoteric’ solvent, but still usage is an order of magnitude behind the petrochemical hydrocarbon solvents [345].

## 11. Conclusions

This review set out to describe the opportunities for renewable platform molecules to be integrated into the petrochemical industry, as well as to indicate some probable market barriers. Whereas other commentaries on this subject have adopted a position of advocating chemically distinct biorefinery products, this work has looked to conformity with the established chemical product production chains. It has been found on reflection that it is mostly not suitable to generate the conventional base chemicals from biomass as a default option. Instead the larger set of intermediate chemicals presently derived from the base chemicals can be readily accessed from biomass without operating counter current to the recognised value chain. For example propanediols are best synthesised directly from glycerol, not proceeding via propylene. Furthermore the primary products of the petrochemical industry are also often the end-points of fermentations (1-butanol, acetone, *etc*.).

**Table 5:** The contrast between the fossil-derived base chemicals and the most promising bio-based intermediates with respect to producing chemicals for the solvent market.

Molecular Size	Fossil Derived Base Chemicals	Bio-Based Platform Molecules
C_1_	Syngas	Bio-syngas
		Methanol
C_2_	Ethylene	Ethanol
		Bio-ethylene
C_3_	Propylene	Glycerol
		Acetone
		Lactic acid
C_4_	Butene(s)	1-Butanol
	Butadiene	Succinic acid
		Isobutanol
C_5_	n/a	Levulinic acid
		Furfural
C_6_	Benzene	HMF
		Levoglucosenone
C_7_	Toluene	n/a
C_8_	Xylene(s)	n/a

This does not apply to all circumstances however. Bio-based syngas and a stronger reliance on ethylene made from bio-ethanol (and its subsequent conversion into propylene and butene) have been envisaged as becoming increasing important to the solvent market, but only as an indirect consequence of the feedstock demand generated by the polyolefin plastics industry (Table 5). Without that driver probably only direct ethanol derivatives would be economically viable bio-based solvent products (e.g., ethyl esters). Beyond C_4_ molecules, the bio-based intermediates that attract most interest are levulinic acid, furfural, and 5-(hydroxymethyl)furfural (HMF) [7,8]. Furfural is required in the synthesis of 2-MeTHF [161], and its hydrolysed derivative 1,2-pentanediol [340]. The other two molecules, HMF and its downstream product levulinic acid, do not have a significant role in solvent synthesis. One exception is γ-valerolactone, which is made from levulinic acid, and can itself be reduced to 2-MeTHF [346]. As an alternative C_6_ intermediate to HMF, levoglucosenone has already been mentioned [347].

**Figure 16:**
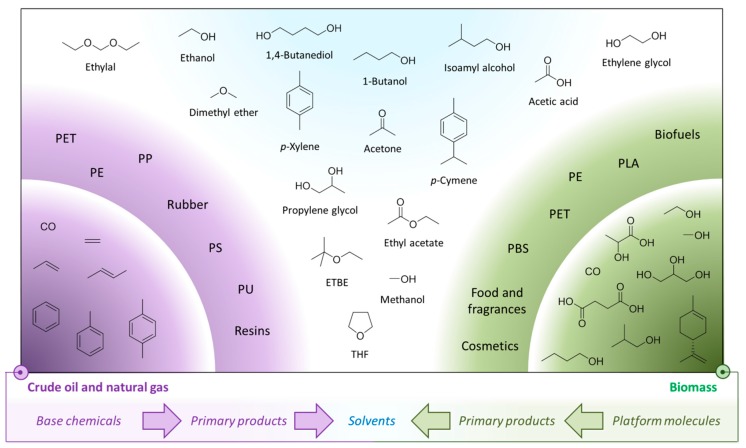
Solvents common to the petrochemical industry and the emerging bio-based economy.

While it is not necessary for biorefineries to strictly conform to the petrochemical precedent, the oil refinery and the bio-refinery should be viewed not as opposing philosophies. Instead the two approaches harmonise at the chemical intermediate phase of the value chain. This is helpful for the bio-based solvent sector because solvents are in many cases intermediates and by-products, or serve some other primary purpose (Figure 16). The earlier and more widespread biomass becomes within chemical manufacturing operations, the greater the acceleration of bio-based solvent uptake should become. Novel bio-based product streams also feed into the chemical intermediates market and will in time complement and invigorate the bio-based solvent market.
